# ATDC induces an invasive switch in KRAS-induced pancreatic tumorigenesis

**DOI:** 10.1101/gad.253591.114

**Published:** 2015-01-15

**Authors:** Lidong Wang, Huibin Yang, Ethan V. Abel, Gina M. Ney, Phillip L. Palmbos, Filip Bednar, Yaqing Zhang, Jacob Leflein, Meghna Waghray, Scott Owens, John E. Wilkinson, Jayendra Prasad, Mats Ljungman, Andrew D. Rhim, Marina Pasca di Magliano, Diane M. Simeone

**Affiliations:** 1 Department of Surgery,; 2 Translational Oncology Program,; 3 Department of Pediatrics,; 4 Department of Internal Medicine,; 5 Department of Pathology,; 6 Department of Laboratory Animal Medicine,; 7 Department of Radiation Oncology,; 8 Department of Molecular and Integrative Physiology,; 9 Department of Cell and Developmental Biology, University of Michigan, Ann Arbor, Michigan 48109, USA

**Keywords:** pancreatic ductal adenocarcinoma, metastasis, epithelial–mesenchymal transition

## Abstract

The initiation of pancreatic ductal adenocarcinoma (PDA) is linked to activating mutations in *KRAS*. However, in PDA mouse models, expression of oncogenic mutant KRAS during development gives rise to tumors only after a prolonged latency or following induction of pancreatitis. Here we describe a novel mouse model expressing ataxia telangiectasia group D complementing gene (ATDC, also known as TRIM29 [tripartite motif 29]) that, in the presence of oncogenic KRAS, accelerates pancreatic intraepithelial neoplasia (PanIN) formation and the development of invasive and metastatic cancers. We found that ATDC up-regulates CD44 in mouse and human PanIN lesions via activation of β-catenin signaling, leading to the induction of an epithelial-to-mesenchymal transition (EMT) phenotype characterized by expression of Zeb1 and Snail1. We show that ATDC is up-regulated by oncogenic Kras in a subset of PanIN cells that are capable of invading the surrounding stroma. These results delineate a novel molecular pathway for EMT in pancreatic tumorigenesis, showing that ATDC is a proximal regulator of EMT.

## Abstract (precis)

Wang et al. describe a mouse model expressing ATDC (TRIM29) that, in the presence of oncogenic KRAS, accelerates PanIN formation and the development of invasive and metastatic pancreatic cancers. ATDC up-regulates CD44 in mouse and human PanIN lesions via activation of β-catenin signaling, leading to the induction of an epithelial-to-mesenchymal transition (EMT) phenotype. ATDC is up-regulated by oncogenic Kras in a subset of PanIN cells that are capable of invading the surrounding stroma.

Pancreatic ductal adenocarcinoma (PDA) is a highly lethal disease often diagnosed in an advanced state when there are little/no effective therapies. It has the worst prognosis of any major malignancy (<5% 5-yr survival) and is predicted to become the second leading cause of cancer death in the United States by 2020 (Rahib et al. 2014). One of the hallmarks of PDA is extensive tumor invasion and early systemic dissemination. In rare patients who present with localized disease on clinical imaging at the time of diagnosis, 70% will die from recurrent disease despite seemingly curative surgical resection (Neoptolemos et al. 2004), suggesting that subclinical spread may occur at the earliest stages of PDA (Sakorafas and Sarr 2003). These clinical data are supported by a recent study using lineage tracing in a genetically engineered mouse model (GEMM) of PDA in which lineage-marked pancreatic epithelial cells were found to undergo epithelial–mesenchymal transition (EMT), invade the stroma, and enter the bloodstream when conventional histology only revealed preinvasive pancreatic intraepithelial neoplasia (PanIN) (Rhim et al. 2012). Thus, the metastatic cascade may be initiated early in the natural history of PDA. However, the molecular underpinnings of early vascular spread are unclear, hindering the development of effective treatments for this disease.

In a previous study, we reported that ataxia telangiectasia group D complementing gene (ATDC) is highly expressed in almost 90% of human PDAs, suggesting that it has an oncogenic function in the disease (Logsdon et al. 2003). ATDC, located on chromosome 11q23 and also known as TRIM29, is a member of the tripartite motif (TRIM) protein family. TRIM family proteins have been implicated in development and growth and in human diseases, including HIV infection (Nisole et al. 2005) and leukemia (Wolf and Goff 2007). We showed that overexpression of ATDC leads to transformation and acquisition of invasive properties in PDA cell lines through β-catenin stabilization and Wnt pathway activation (Wang et al. 2009). ATDC has also been shown to bind p53 and antagonize p53-mediated nuclear functions (Yuan et al. 2010). To further explore the biological function of ATDC in PDA initiation and progression, we generated GEMMs of pancreatic cancer expressing both oncogenic KRAS and ATDC to examine in greater detail the contribution of ATDC to tumor initiation and progression. Our results suggest that ATDC may promote tissue invasion and vascular spread early in the course of the disease by inducing EMT.

## Results

### Generation and phenotypic characterization of ATDC transgenic (TG) mice

**Figure 1.**
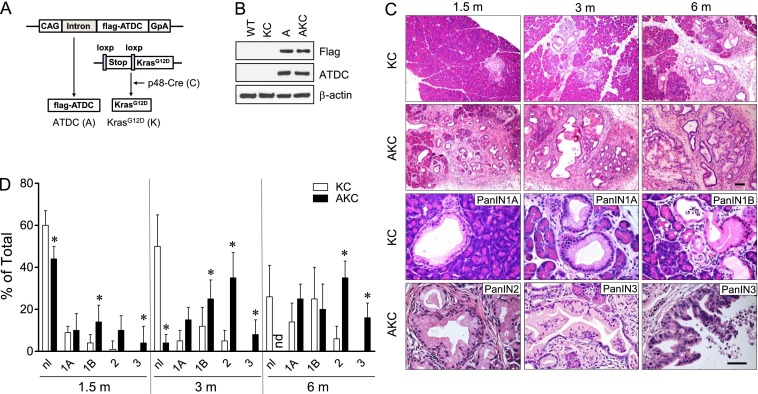
ATDC accelerates PanIN progression in KC mice. (*A*) Schematic of the ATDC TG construct used to generate CAG-ATDC mice. (A) ATDC; (K) LGL-Kras^G12D^; (C) p48-Cre; (CAG) CAG promoter; (GpA) β-globin polyA. (*B*) Representative Western blot using Flag and ATDC antibodies showing ATDC transgene expression in ATDC (A) and AKC (ATDC; LGL-Kras^G12D^; p48-Cre) mice but not in wild-type (WT) and KC (LGL-Kras^G12D^; p48-Cre) mice. (*C*) Representative images of PanIN lesions in 1.5-, 3- and 6-mo-old KC or AKC mice. Bars: *top* panel, 100 µm; *bottom* panel, 50 µm. (*D*) Quantification of PanIN lesions at the indicated time points in KC or AKC mice. Percentages (±SEM) of normal (nl) and PanINs by grade (1A, 1B, 2, 3) in mice at 1.5 mo (*n* = 12), 3 mo (*n* = 15), and 9 mo (*n* = 15) of age. (*) *P* < 0.05 versus KC. (nd) Not detected.

To understand the contribution of ATDC overexpression in pancreatic tumorigenesis, we generated TG mice (FVB/NJ background) with expression of human Flag-tagged ATDC driven by a constitutive cytomegalovirus/chicken β-actin enhancer/promoter (CAG promoter) (Fig. 1A). TG mice were born at the expected Mendelian ratio and were viable, with no gross abnormalities. Flag-ATDC expression was confirmed in the pancreas by Western blotting and immunohistochemical analysis of CAG-ATDC TG mice (Fig. 1B; Supplemental Fig. 1A). Histopathological analysis of pancreata from CAG-ATDC mice at early time points (1–5 mo) showed normal-appearing exocrine glandular components and islets, whereas at later time points (6–12 mo), pancreata exhibited acinar atypia (Supplemental Fig. 1B). However, over the course of 12 mo, no PanIN lesions were detected in any CAG-ATDC TG mice. CAG-ATDC TG mice did not have alterations in organ size or cell death rate; however, a slight increase in cell proliferation, compared with control littermates, was observed (Supplemental Fig. 1D). A subset of these mice (30%) developed invasive bladder tumors within 12 mo, limiting more prolonged analysis due to institutional laboratory animal safety committee concerns.

Because both *KRAS* mutation and ATDC overexpression occur in most human PDAs and oncogenic KRAS is needed for PDA development, we crossed CAG-ATDC mice with p48-Cre;LSL-Kras^G12D^ mice (“KC” mice) (Hingorani et al. 2003) to generate p48-Cre;LSL-Kras^G12D^; CAG-ATDC mice, termed “AKC” mice. In the KC model in which the Kras^G12D^ gene is activated in a tissue-specific manner upon Cre recombination, mice are born with a normal pancreas and gradually develop PanIN lesions at 12 wk of age; a small subset (∼15%) of KC mice progress to invasive PDA with a long latency (>12 mo) (Aguirre et al. 2003; Hingorani et al. 2003, 2005). As previously described (Hingorani et al. 2003), mice harboring either the LSL-Kras^G12D^ or p48-Cre allele alone showed no pancreatic abnormalities over the 12-mo observation period (data not shown). KC mice developed PanIN1A lesions at 1.5∼3 mo of age (Fig. 1C,D), with progression to PanIN1B or PanIN2 lesions at 3–6 mo of age (Fig. 1C,D; Supplemental Fig. 2A). In contrast, at 1.5 mo of age, pancreata of AKC mice presented with PanIN1B and PanIN2 lesions surrounded by a desmoplastic reaction comprised of fibroblasts/stellate cells, infiltrating inflammatory cells, and collagen deposition (Fig. 1C,D; Supplemental Fig. 2A). At 3–6 mo of age, AKC mice had a greater number of PanIN lesions (Supplemental Fig. 1C) and contained more advanced PanIN lesions compared with KC mice, including PanIN3 lesions (Fig. 1C,D). AKC PanIN lesions exhibited characteristic histological changes, including conversion of ductal epithelial cells to a columnar phenotype with mucin accumulation (Alcian blue staining) and stromal collagen deposition (positive trichrome staining) (Supplemental Fig. 2B). These data show that ATDC overexpression dramatically accelerates PanIN initiation and progression in the setting of an oncogenic *Kras* mutation.

**Figure 2.**
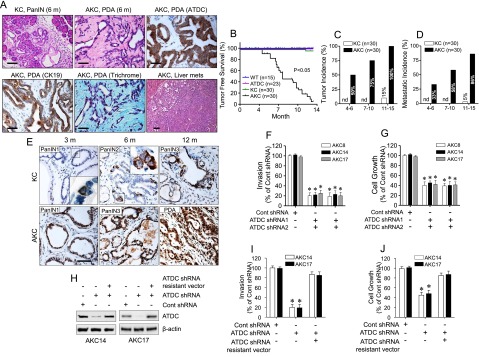
ATDC promotes invasive and metastatic disease in KC mice. (*A*) Representative images of PanIN lesions, PDAs, and liver metastases in KC and AKC mice. Immunohistochemistry staining for ATDC, CK19 expression, and Gomori trichrome staining for interstitial collagen in AKC mice. Bar, 50 µm. (*B*) Kaplan-Meier curves depicting survival rates for wild-type (WT), CAG-ATDC (ATDC), KC, and AKC mice. (*C*,*D*) Quantification of tumor incidence (*C*) or metastatic incidence (*D*) at the indicated time periods in KC (white bars) or AKC (black bars) mice. (nd) Not detected. (*E*) Representative images depicting ATDC expression in KC and AKC mice at the indicated ages. Bar, 50 µm. (*F*,*G*) Invasion assays (*F*) and cell growth assays (*G*) in AKC8, AKC14, and AKC17 cells expressing control shRNA, ATDC shRNA1, or ATDC shRNA2. (*H*) Representative Western blot showing ATDC expression in AKC14 and AKC18 cell lines with or without control shRNA (Cont shRNA), ATDC shRNA, or ATDC shRNA-resistant vector expression. β-Actin served as a control. (*I*,*J*) Invasion assays (*I*) and cell growth assays (*J*) in AKC14 and AKC17 cells expressing control, ATDC shRNA, or ATDC shRNA-resistant vector. For *F*,*G*, *I* and *J*, data represent the mean ± SEM. (*) *P* < 0.05 versus control shRNA; *n* = 4.

We then followed a cohort of 30 KC and 30 AKC mice. In the KC group, we identified only three invasive tumors, which developed from 12 to 14 mo of age. In contrast, AKC mice developed invasive PDAs as early as 4 mo of age, with 100% of the mice developing PDA at 12 to 14 mo of age (Fig. 2A; Supplemental Fig. 2A,C). Histologically, pancreatic adenocarcinomas that developed in AKC mice were defined by the presence of neoplastic glandular tissue and dense fibrous stroma, with the majority of tumors well or moderately differentiated, with occasional regions of sarcomatoid change (Table 1; Supplemental Fig. 2D; Supplemental Table 2).

**Table 1.**
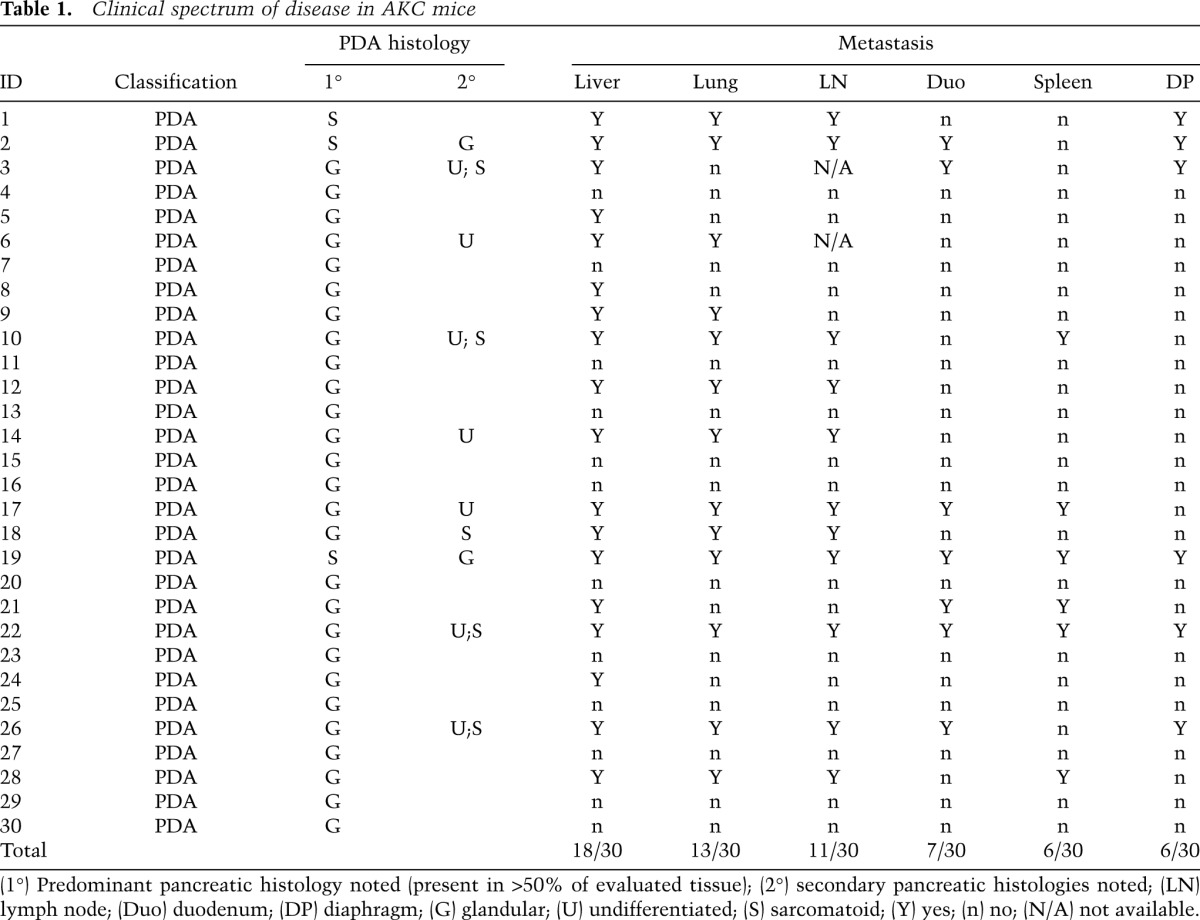
Clinical spectrum of disease in AKC mice

The ductal phenotype of the tumors was confirmed with positive staining for the ductal markers CK-19 (Fig. 2A; Supplemental Fig. 2B) and DBA lectin (data not shown). AKC mouse tumors possessed a striking resemblance to human PDA, with invasion and metastatic spread to lymph nodes, lungs, the liver, and other sites (Fig. 2A; Table 1; Supplemental Fig. 2C), supporting the relevance of this model to human disease. At 7–10 mo of age, invasive cancers were present in >75% of AKC mice, with 58% of the PDA-bearing mice developing overt metastatic disease with abdominal distension due to the presence of malignant ascites (Fig. 2C,D). This disease burden was reflected by the shortened life span of AKC mice compared with KC mice, with an average survival in the studied cohort of 7 mo (Fig. 2B).

We assessed the genetic status of three of the most commonly mutated genes in human PDA and drivers of PDA in various GEMMs (*p53*, *p16*^*Ink4*^, and *Smad4)* in primary AKC tumors and three different low-passage cell lines (AKC8, AKC14, and AKC17) derived from different AKC tumors. Primary tumors from AKC mice contained wild-type *p53*, *p16*^*Ink4*^, and *Smad4* genes (Supplemental Table 1). Additional experiments demonstrated induction of p53 and p21^CIP1^ in response to the Mdm2 antagonist nutlin-3 in the AKC8 and AKC17 cell lines derived from AKC tumors and Smad4 nuclear translocation after TGFβ treatment, indicating wild-type Smad4 and p53 function (Supplemental Fig. 3). Therefore, overexpression of ATDC in the absence of additional driver mutations was sufficient to induce tumorigenesis in KC mice.

We next hypothesized that the level of ATDC correlated with neoplastic progression and metastasis. Analysis of pancreata harvested from KC and AKC mice at different ages revealed the presence of occasional ATDC-expressing cells in KC mice even in early PanIN lesions, with a gradual increase in the numbers of ATDC-expressing cells as PanIN lesions progressed to invasive carcinomas (Fig. 2E; Supplemental Fig. 4A,B). Quantitative RT–PCR (qRT–PCR) analysis of harvested pancreata from 3-, 6-, and 8-mo-old KC and AKC mice confirmed a gradual increase in ATDC expression during PanIN–PDA progression, albeit at levels lower than those observed in AKC mice (Supplemental Fig. 4B).

To determine whether oncogenic Kras was capable of inducing ATDC expression, we measured ATDC levels in both a mouse PDA cell line (derived from Kras^G12D^;Trp53^R172H^;p48-Cre mice [KPC mice]) and human (Capan2) pancreatic cancer cell lines expressing control or ATDC shRNA lentiviral vectors. Kras knockdown significantly decreased ATDC expression (Supplemental Fig. 5A,B). To determine whether reintroduction of ATDC was able to rescue the effect of Kras knockdown, we transfected ATDC into both KPC and Capan2 cells expressing Kras shRNA lentiviral vectors to restore ATDC expression (Supplemental Fig. 5C). In both cell lines, reintroduction of ATDC restored the effects of Kras knockdown on cell proliferation and invasion in vitro (Supplemental Fig. 5D,E).

### ATDC promotes cell invasion and an EMT phenotype in vivo

Given the frequency of invasive and metastatic disease in AKC mice, we hypothesized that ATDC overexpression might induce EMT. We first used cell lines derived from primary PDAs from AKC mice (AKC8, AKC14, and AKC17) and confirmed elevated ATDC expression in these cell lines compared with the normal pancreas (Supplemental Fig. 6A). To determine the role of ATDC in invasion, we generated AKC cell lines expressing scrambled control shRNA or ATDC-targeting shRNA1 and shRNA2 (Supplemental Fig. 6B). The expression of ATDC shRNA1 and shRNA2 significantly inhibited invasion in AKC8, AKC14, and AKC17 cells compared with controls (Fig. 2F), suggesting that ATDC regulates pancreatic cancer cell invasion. ATDC knockdown also decreased cellular proliferation in AKC cell lines (Fig. 2G), although the effect on proliferation was less pronounced than that observed in the invasion assays. To validate the specificity of ATDC knockdown on the observed effect on invasion, we generated an ATDC shRNA-resistant expression vector using the ATDC shRNA1 targeting sequence (Fig. 2H; Supplemental Fig. 6C). Introduction of the ATDC shRNA-resistant vector into AKC14 and AKC17 cells expressing ATDC shRNA rescued the inhibition of invasion and cell proliferation induced by ATDC shRNA, indicating the specificity of these constructs (Fig. 2I,J).

**Figure 3.**
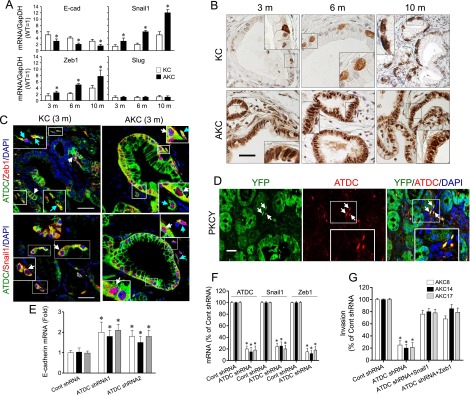
ATDC up-regulates Zeb1 and Snail1 expression and induces EMT in vivo. (*A*) qRT–PCR analysis of EMT genes in PanIN or PDA samples from KC (white bars) and AKC (blue bars) mice at the indicted ages. (m) Month. GapDH was used as a loading control. All values were normalized to pancreatic samples from age-matched wild-type (WT) littermates, which were set to 1. (*) *P* < 0.05, AKC versus KC; *n* = 3. (*B*) Immunohistochemical analysis of Snail1 expression in pancreata harvested from KC and AKC mice at the indicated ages. *Insets* indicate magnified views of staining patterns. Bar, 50 µm. (*C*) Coimmunofluorescent staining of ATDC (green), Zeb1 or Snail1 (red), and DAPI (blue) in PanIN lesions from 3-mo-old KC or AKC mice. *Insets* indicate magnified views of staining patterns. White arrows indicate nuclear staining of Zeb1 and Snail1. Light-blue arrows indicate ATDC/Zeb1- or ATDC/Snail1- double-positive cells located in the stroma surrounding PanIN lesions. Bar, 50 µm. (*D*) Coimmunofluorescent staining of ATDC and GFP in pancreatic tissues from PKCY mice shows colocalization (yellow) of individual epithelial YFP-expressing (green) cells and ATDC-expressiong (red) cells. Arrows indicate mesenchymal-appearing ATDC-positive epithelial cells that have extravasated into the stroma surrounding PanIN lesions. Bar, 10 µm. (*E–G*) qRT–PCR analysis of E-cadherin (*E*) and ATDC, Snail1, and Zeb1 expression (*F*) or invasion assays (*G*) using AKC8, AKC14, and AKC18 cell lines expressing control or ATDC shRNA with or without transfection of Snail1 or Zeb1 expression vectors (*G*). Data represent the mean ± SEM, expressed as a percentage of control shRNA. (*) *P* < 0.05 versus control shRNA; *n* = 4.

To assess the impact of ATDC on EMT, we examined the expression of EMT markers in PanIN lesions or PDA samples obtained from KC and AKC mice at the indicated ages. During PanIN-to-PDA progression, the expression of Snail1 and Zeb1 increased and E-cadherin decreased in both KC and AKC mice, although the increases of Snail1 and Zeb1 observed in AKC mice were more significant than those seen in KC mice (Fig. 3A). To further determine the effect of ATDC on the EMT phenotype, we examined cellular morphology in KPC cells, chosen to examine the impact of ATDC alteration in the endogenous setting. Knockdown of ATDC in KPC cells not only increased E-cadherin and decreased Snail1 and Zeb1 expression (Supplemental Fig. 7A–C) but also resulted in a pronounced change in cellular morphology from a more mesenchymal phenotype to a more epithelial phenotype (Supplemental Fig. 7D). To verify these findings in vivo, we performed immunohistochemical staining to assess expression of EMT markers in KC and AKC pancreata harvested at different ages. Consistent with our qRT–PCR data, AKC pancreatic tissues revealed increased Zeb1 and Snail1 and decreased E-cadherin protein expression during PanIN-to-PDA progression compared with similarly staged KC samples (Fig. 3B; Supplemental Fig. 8). Immunofluorescent staining revealed colocalization of ATDC with Zeb1 or Snail1 in a small subset of cells in low-grade PanIN lesions from KC mice, indicating that these ATDC-expressing cells have undergone EMT (Fig. 3C; Supplemental Fig. 9A,C). Quantitation of Zeb1/ATDC or Snail1/ATDC double-positive cells and their percentage of total PanIN cells is shown in Supplemental Table 6. Interestingly, some isolated, mesenchymal-looking double-positive cells were present in the stroma surrounding early PanIN lesions in KC and AKC mice (Fig. 3C; Supplemental Fig. 9A,C). Similar results were seen in human PanIN lesions (Supplemental Fig. 9B,D). There were increased numbers of Zeb1/ATDC- or Snail1/ATDC-expressing cells present in low-grade PanIN lesions of AKC mice and disseminated in the stroma, suggesting an intensified EMT phenotype in AKC mice (Fig. 3C; Supplemental Fig. 8; Supplemental Table 6). Of note, there was no evidence of EMT marker expression in the pancreata of KC or AKC mice prior to PanIN lesion development (Supplemental Fig. 8A,B).

To determine whether stromal ATDC-positive cells were epithelial cells that had undergone EMT, we analyzed a lineage-labeled mouse model of PDA (PKCY) in which all pancreatic epithelial-derived cells are marked with YFP at birth (Rhim et al. 2012). In PKCY mice that contained only PanIN lesions and no invasive carcinoma on histology, we indeed observed ATDC expression within YFP^+^ lineage-labeled pancreas epithelial-derived cells that had delaminated into the stroma (Fig. 3D). Thus, ATDC is expressed in a subset of pancreatic epithelial-derived cells that undergo EMT in vivo and acquire invasive capability prior to the formation of invasive tumors on histology.

### ATDC induces EMT by CD44 up-regulation

We next sought to determine the mechanism by which ATDC induces EMT in pancreatic neoplastic cells. We compared primary PDA cell lines AKC8, AKC14, and AKC17 that were transfected with ATDC-specific and scrambled shRNA lentiviral constructs. Knockdown of ATDC led to increased E-cadherin and decreased expression of Snail1 and Zeb1 mRNAs (Fig. 3E,F) with no effect on GAPDH gene expression (Supplemental Fig. 10A). To determine whether Snail1 and Zeb1 induction by ATDC was responsible for the invasion-promoting properties of ATDC, we performed invasion assays. Knockdown of ATDC significantly decreased invasiveness of AKC8, AKC14, and AKC17 cells, and this effect was completely rescued by transfection with Snail1 or Zeb1 expression vectors (Fig. 3G), which restored expression of Snail1 and Zeb1 to control levels (Supplemental Fig. 10B,C). Of note, knockdown of Snail1 or Zeb1 did not alter ATDC gene expression (Supplemental Fig. 11A,B,E,F). Thus, ATDC-dependent acquisition of an invasive phenotype by PDA cells is mediated through the up-regulation of the EMT drivers Snail1 and Zeb1.

**Figure 4.**
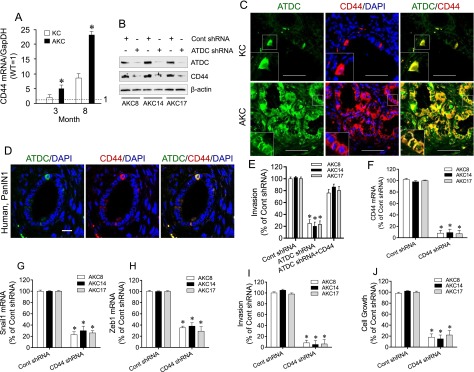
Up-regulation of CD44 by ATDC enhances expression of the EMT genes Snail1 and Zeb1 and invasiveness of pancreatic cancer cells. (*A*) qRT–PCR analysis of CD44 gene expression in PanIN lesions or PDA samples from KC (white bars) or AKC (black bars) mice. GapDH was used as the control. All values were normalized to pancreatic samples from age-matched wild-type (WT) littermates, which were set to 1. (*) *P* < 0.05 AKC versus KC; *n* = 3. (*B*) Western blot analysis of CD44 expression in AKC8, AKC14, and AKC18 cells expressing control (Cont) or ATDC shRNA. (*C*) Coimmunofluorescent staining of ATDC (green), CD44 (red), and DAPI (blue) in pancreatic sections from KC and AKC mice (3 mo of age). Bar, 50 µm. Yellow shows colocalization of ATDC and CD44. (*D*) ATDC (green), CD44 (red), and DAPI (blue) coimmunofluorescence in human PanIN1 lesions. Yellow shows colocalization of ATDC and CD44. Bar, 10 µm. (*E*) Invasion assays in AKC8, AKC14, and AKC18 cells expressing control or ATDC shRNA. The introduction of the CD44 expression vector in ATDC shRNA-expressing cells rescued the inhibitory effect of ADTC shRNA on invasion. (*F–H*) qRT–PCR analysis of CD44 (*F*), Snai1l (*G*), and Zeb1 (*H*) in AKC8, AKC14, and AKC18 cells expressing control or CD44 shRNA. (*I*,*J*) Invasion (*I*) and growth (*J*) assays in AKC8, AKC14, and AKC18 cells expressing control or CD44 shRNA. Data are presented as the mean ± SEM. (*) *P* < 0.05 versus control shRNA. *n* = 4.

We next sought to determine how ATDC induces EMT in PanINs or PDA. In addition to our data showing up-regulation of CD44 by ATDC, it has previously been shown that CD44 is a switch for EMT and that YFP^+^ pancreatic epithelial cells undergoing EMT in vivo in the KPCY model expressed CD44 (Kim et al. 2008; Afify et al. 2009; Brown et al. 2011; Jung et al. 2011; Rhim et al. 2012). Furthermore, many studies have shown that knockdown of CD44 or the use of CD44-blocking antibodies inhibited EMT and invasion (Kim et al. 2008; Afify et al. 2009; Craig et al. 2009; Bhat-Nakshatri et al. 2010; Brown et al. 2011; Provenzano et al. 2012; Li et al. 2014). Using qRT–PCR and immunostaining analysis, we observed that CD44 was significantly up-regulated in PanIN lesions and PDA arising in AKC mice compared with KC lesions, while CD44 was not significantly expressed in age-matched wild-type mice, ATDC mice, or KC and AKC littermates prior to PanIN formation (Fig. 4A; Supplemental Fig. 12A). While CD44 expression was observed in low-grade PanIN lesions and gradually increased during PanIN-to-PDA progression in KC mice, CD44 expression was significantly lower in KC mice compared with AKC mice (Fig. 4A; Supplemental Fig. 12A). Knockdown of ATDC significantly decreased CD44 protein expression in AKC8, AKC14 and AKC17 cells (Fig. 4B). To further determine the association between ATDC and CD44, we performed coimmunofluorescent staining. As shown in Figure 4C, individual CD44/ATDC-coexpressing epithelial cells were observed in low-grade PanIN lesions from KC mice (3 mo of age). Coexpression of ATDC and CD44 was also observed in PanIN lesions from AKC mice (3 mo of age); however, these lesions exhibited a much greater number of ATDC/CD44-double-positive cells (Fig. 4C). Furthermore, the majority of ATDC-positive cells colocalized with CD44-positive cells in PDA from both KC (12-mo-old) and AKC (5-mo-old) mice (Supplemental Fig. 12B). Importantly, colocalization of ATDC- and CD44-positive epithelial cells was also observed in low-grade human PanIN lesions (Fig. 4D).

Knockdown of CD44 in PDA cells not only effectively decreased expression of the EMT markers Snail1 and Zeb1 (Fig. 4F–H) but also significantly attenuated invasion and cell growth in AKC8, AKC14, and AKC17 cells (Fig. 4I,J). Knockdown of ATDC in PDA cells led to a significant decrease in CD44 expression. Furthermore, invasive capability was rescued in PDA cell lines with ATDC knockdown when cells were transfected with a CD44 expression vector (Fig. 4E; Supplemental Fig. 12C). Knockdown of Snail1 and Zeb1 expression by shRNA did not impact ATDC or CD44 mRNA levels (Supplemental Fig. 11A,B,D–F,H). Importantly, we were able to validate these findings using low-passage cancer cell lines (UM18 and UM59) derived from human primary PDA samples, where silencing of ATDC significantly inhibited CD44, Snail1, and Zeb1 expression (Supplemental Fig. 12D). These data suggest that ATDC triggers an invasive EMT phenotype in premalignant pancreatic epithelium and PDA via CD44 up-regulation.

### Activation of β-catenin is required for ATDC-mediated induction of Cd44 and EMT

**Figure 5.**
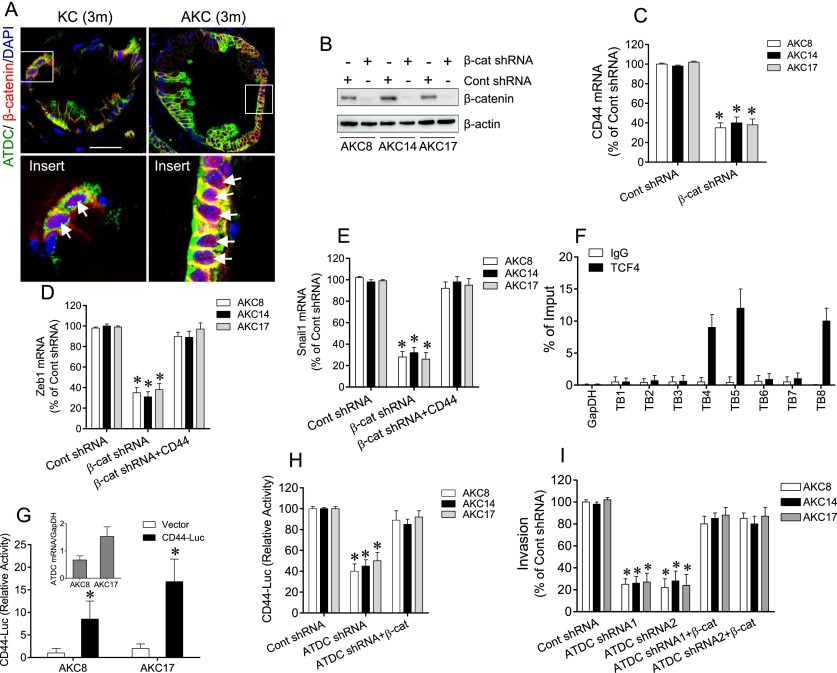
Activation of β-catenin by ATDC increases CD44 transcriptional activity via TCF4 binding to the CD44 promoter. (*A*) Coimmunofluorescent staining of ATDC (green), β-catenin (red), and DAPI (blue) in PanIN lesions from 3-mo-old KC or AKC mice. *Insets* indicate magnified views of staining patterns. White arrows indicate nuclear staining of β-catenin. Bar, 50 µm. (*B*) Representative Western blot showing β-catenin expression in AKC8, AKC14, and AKC18 cells expressing control (Cont) or β-catenin (β-cat) shRNA. β-Actin served as a loading control. (*C–E*) qRT–PCR analysis of CD44 (*C*), Zeb1 (*D*), and Snail1 (*E*) mRNA expression in AKC8, AKC14, and AKC18 cells expressing control or β-catenin shRNA. In *D* and *E*, transfection with the CD44 expression vector in β-catenin shRNA-expressing AKC8, AKC14, and AKC18 cells restored Zeb1 (*C*) and Snail1 (*D*) to control shRNA levels. (*F*) ChIP assays were performed using an anti-TCF4 antibody (or normal IgG control) and analyzed by real-time PCR in AKC17 cells to identify TCF4-binding sites to the CD44 promoter. Binding to regions in the CD44 promoter containing TCF4-binding motifs (TB1–8) was detected using specific primers. The percentage of enriched binding was calculated over input DNA (*n* = 3). The GapDH promoter region was used as negative control. (*G*) qRT–PCR analysis of ATDC gene expression in AKC8 and AKC17 cells. (*Insert*) CD44 luciferase reporter activity (CD44-Luc) correlated with ATDC gene expression levels in AKC8 and AKC17 cells. (*H*) CD44 luciferase reporter gene activity in AKC8, AKC14, and AKC17 cells expressing control or ATDC shRNA. Transfection of a vector expressing β-catenin in the ATDC shRNA-expressing cells rescued that inhibitory effect of ATDC shRNA on CD44 luciferase reporter activity. (*I*) Invasion assays using AKC8, AKC14, and AKC18 cell lines expressing control or ATDC shRNA with or without transfected β-catenin expression vectors. Data represent mean ± SEM. (*) *P* < 0.05 versus control shRNA; *n* = 4.

We previously demonstrated that overexpression of ATDC promotes activation of β-catenin signaling by binding to and stabilizing Dvl2, resulting in the release of β-catenin from the destruction complex in human PDA cells (Wang et al. 2009). Activation of the β-catenin signaling pathway has been shown to induce EMT (Sanchez-Tillo et al. 2011). Furthermore, it has been shown that CD44 expression was completely abolished in TCF4 knockout mice (Wielenga et al. 1999). Therefore, we hypothesized that ATDC-mediated induction of CD44 expression in AKC mice might require activation of β-catenin/TCF signaling. We did observe that β-catenin expression was significantly increased in PanIN and PDA lesions in AKC mice, with lower degrees of expression in CAG-ATDC mice and AKC mice prior to neoplastic transformation (Supplemental Fig. 13). Furthermore, we observed colocalization of β-catenin and ATDC in cells within low-grade PanIN lesions from KC mice, with some evidence of nuclear β-catenin staining (Fig. 5A, left panels). The β-catenin pathway was strongly activated in PanIN lesions in AKC mice, with enhanced β-catenin nuclear staining in ATDC/β-catenin-double-positive cells (Fig. 5A, right panels). To verify that overexpression of ATDC promotes activation of β-catenin in AKC cells in a manner similar to that seen in human PDA cells, we performed coimmunoprecipitation experiments and demonstrated that ATDC interacted with Dvl-2 in AKC14 and AKC17 cells, while knockdown of ATDC significantly inhibited the interaction of ATDC and Dvl-2 as well as β-catenin expression (Supplemental Fig. 14A). A strong correlation between ATDC and β-catenin expression levels was observed in AKC, KPC, and KC PDA cells (Supplemental Fig. 14B).

To prove that β-catenin served as a critical link between ATDC and CD44 expression, we knocked down β-catenin in AKC cells (Fig. 5B), and this resulted in a significant reduction of CD44 (Fig. 5C), Zeb1 (Fig. 5D), and Snail1 (*P* < 0.05 [*] vs. control shRNA) (Fig. 5E). These results support our hypothesis that ATDC promotes induction of CD44 and EMT by activating the β-catenin signaling pathway.

To determine that CD44 is a critical mediator of β-catenin-induced EMT, we transfected AKC8, AKC14, and AKC17 cells expressing β-catenin shRNA with a CD44 expression vector and found that this reversed the inhibitory effect of β-catenin knockdown on Zeb1 and Snail1 gene expression (Fig. 5D,E). Of note, alteration of Snail1 and Zeb1 expression did not impact β-catenin gene expression levels (Supplemental Fig. 11C,G). Coimmunofluorescent staining in AKC17 cells expressing control, ATDC, or β-catenin shRNAs revealed that knockout of ATDC or β-catenin significantly decreased both CD44 and Snail1 expression in AKC17 cells (Supplemental Fig. 15).

### CD44 is up-regulated by ATDC via β-catenin-mediated activation of the CD44 promoter

To understand the mechanism by which activated β-catenin/TCF signaling induces CD44 expression to promote PanIN-to-PDA progression, we examined the CD44 promoter region for potential TCF4-binding sites. Using previously described TCF4-binding sequences (Jho et al. 2002; Blauwkamp et al. 2008) as well as the Transcription Element Search System (TESS), we found several noncanonical TCF4-binding sites [AGAA(T)AT] upstream (−1500 base pairs [bp]) of the CD44 transcription start site (TSS) (Supplemental Fig. 16A; Blauwkamp et al. 2008). To determine whether TCF4 interacted with the CD44 promoter region, we performed quantitative chromatin immunoprecipitation (ChIP) assays using a TCF4-specific antibody in AKC17 cells. We designed primers flanking eight putative TCF4-binding sites ∼1.5 kb upstream of the TSS of the CD44 gene. Immunoprecipitation of TCF4 resulted in enrichment of three highly conserved consensus sites in CD44 (TB4, TB5, and TB8) (Fig. 5F). These findings support the hypothesis that β-catenin/TCF4 promotes CD44 expression by binding to the CD44 promoter.

To explore the effects of ATDC-mediated activation of β-catenin on CD44 transcription, we used a CD44 promoter/luciferase reporter construct containing the TCF4-binding sites TB4, TB5, and TB8. As shown in Figure 5G, CD44 promoter reporter activity correlated with ATDC expression levels in AKC17 and AKC8 cells. Furthermore, knockdown of ATDC effectively attenuated CD44 promoter reporter activity in AKC cells (Fig. 5H). Transfection of the ATDC knockdown cells with a β-catenin expression vector (Supplemental Fig. 16B) resulted in a complete rescue of CD44 promoter reporter activity (Fig. 5H). Furthermore, overexpression of β-catenin effectively rescued impaired invasion (Fig. 5I) and growth (Supplemental Fig. 14C) in AKC cells with ATDC knockdown compared with control cells. These findings show that ATDC promotes CD44 gene activation via the activation of the β-catenin signaling pathway and that this signaling network is critical for mediating ATDC’s effects on proliferation and invasion.

### Conditional overexpression of ATDC in the pancreatic epithelium of KC mice recapitulates the phenotype of AKC mice

**Figure 6.**
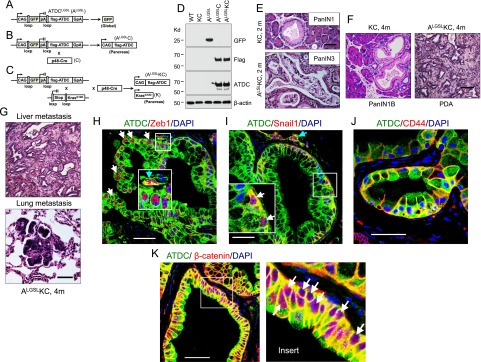
Pancreas-specific overexpression of ATDC drives an invasive and metastatic phenotype in KC mice. Breeding strategy to generate the A^LGSL^;LSL-Kras^G12D^;p48-Cre (A^LGSL^KC) mice. (*A*) Schematic of the ATDC (A^LGSL^) construct. (CAG) CAG promoter; (GpA) β-globin polyA. (*B*,*C*) Breeding strategy to generate A^LGSL^C (*B*) and A^LGSL^KC (*C*) mice. (*D*) Representative Western blot showing GFP expression in A^LGSL^ mice and ATDC expression in A^LGSL^C and A^LGSL^KC mice. (*E*) Representative histologic images of the pancreata of KC and A^LGSL^KC mice at 2 mo (m) of age. Bar, 50 µm. (*F*,*G*) Representative images of H&E staining of pancreata from KC and A^LGSL^KC mice at 4 mo of age (*F*) and metastatic lesions present in the liver and lung of 4-mo-old A^LGSL^KC mice (*G*). Bar, 50 µm. (*H–J*) Coimmunofluorescent staining of ATDC (green), Zeb1 (red) (*H*), Snail1 (red) (*I*), and CD44 (red) (*J*), and DAPI (blue) in PanIN lesions from 3-mo-old A^LGSL^KC mice. *Insets* indicate magnified views of staining patterns. White arrows indicate nuclear staining of Zeb1 and Snail1. Light-blue arrows indicate ATDC/Zeb1- or ATDC/Snail1-double-positive cells located in the stroma adjacent to PanIN lesions. Bar, 50 µm. (*K*) Coimmunofluorescent staining of ATDC (green), β-catenin (red), and DAPI (blue) in PanIN lesions from 3-mo-old A^LGSL^KC mice. *Insets* indicate magnified views of staining patterns. White arrows indicate nuclear staining of β-catenin. Bar, 50 µm.

To determine whether the tumorigenic and invasive phenotype associated with ATDC overexpression is due to pancreatic epithelial or stromal ATDC expression, we generated conditional TG mice expressing a Flag-tagged ATDC transgene specifically in the pancreatic epithelium. The tissue-specific ATDC transgene construct (ATDC^LGSL^) was generated by inserting a GFP (G) coding sequence/polyadenylation signal (S) cassette surrounded by two loxP (L) sites between the Flag-tagged ATDC coding sequence and the CAG promoter (Fig. 6A). In the absence of Cre recombinase, no ectopic ATDC expression was noted (Fig. 6B–D; Supplemental Fig. 17). However, in ATDC^LGSL^;p48-Cre (A^LGSL^C) or ATDC^LGSL^;LSL-Kras^G12D^;p48-Cre (A^LGSL^KC) mice (Figs. 6B–D; Supplemental Fig. 17 B,C), pancreatic-specific overexpression of ATDC induced pancreatic atypia in A^LGSL^C mice within 6–10 mo of age (data not shown). To further explore the tumorigenic function of ATDC, we examined the impact of pancreas-specific overexpression of ATDC on the PanIN-to-PDA progression. Consistent with our observations in AKC mice, PanIN3 lesions developed in A^LGSL^KC mice as early as 2 mo of age, but only PanIN1 lesions appeared in age-matched KC mice (Fig. 6E). At 4 mo of age, invasive PDA, which exhibited evidence of distant metastasis, was observed in 11 out of 15 (73%) A^LGSL^KC mice (Fig. 6F,G). The disease spectrum in A^LGSL^KC mice was consistent with AKC mice (Supplemental Table 2). Our data suggest that pancreatic-specific overexpression of ATDC recapitulates the invasive and metastatic phenotype of PDA observed with global ATDC overexpression and highlight the key role of ATDC in the process of pancreatic tumorigenesis.

To confirm that ATDC overexpression in epithelial cells promotes an EMT phenotype and triggers early dissemination of neoplastic epithelial PanIN cells into the stroma, we conducted coimmunofluorescent staining. As in AKC mice, ATDC potently promoted Zeb1 and Snail1 expression in PanIN lesions of A^LGSL^KC mice (Fig. 6H,I). Furthermore, EMT-like double-positive cells expressing either Snail1 or Zeb1 and ATDC were observed in the stroma surrounding PanIN lesions (Fig. 6H,I). Both β-catenin expression and CD44 expression were significantly enhanced and coexpressed with ATDC in A^LGSL^KC mice (Fig. 6J,K). Based on these data, we also conclude that ATDC overexpression in epithelium cells, rather than in stromal cells, is responsible for neoplastic epithelial cells undergoing a gain of migratory and invasive capabilities associated with the EMT phenotype.

## Discussion

**Figure 7.**
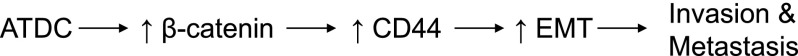
Proposed model for the contribution of ATDC to pancreatic cancer development and progression. Flow chart describing a novel signaling pathway by which ATDC drives EMT and invasion during pancreatic tumorigenesis by β-catenin-mediated up-regulation of CD44.

In this study, we found that progression and metastatic potential of PDA could be greatly enhanced in a Kras^G12D^-driven murine model for pancreatic cancer by the ectopic expression of the human ATDC gene, which is overexpressed in the majority of human PDA samples. A hallmark of this aggressive phenotype included a pronounced increase in the number of cells undergoing EMT, with a concurrent increase in the expression of the EMT-driving factors Snail1, Zeb1, and CD44. We also determined that CD44 was central for promoting the up-regulation of Snail1 and Zeb1 protein expression as well as promoting invasion in vitro. Finally, we determined that CD44 was up-regulated by ATDC via activation of β-catenin/TCF4 signaling, which in turn bound to and activated the CD44 promoter (summarized in Fig. 7). These findings support our earlier findings that ATDC is a potent driver of PDA and suggest that it may play a central role in PanIN-to-PDA progression and metastasis.

Metastatic PDA progresses from a morphologically well-defined preinvasive PanIN lesion by genetic and/or epigenetic alterations (Hruban et al. 2001). Of these alterations, activation of mutations in *KRAS* occurs in almost 95% of PDAs and serves as an early initiating event in pancreatic tumorigenesis (Malumbres and Barbacid 2003). Additionally, mutations in the tumor suppressors *p53*, *p16*^*INK4*^, and *Smad4* occur during the different PanIN stages (Hezel et al. 2006), contributing to the development of more advanced and invasive PDA (Vogelstein and Kinzler 2004). Importantly, these alterations in the tumor suppressor genes are often necessary for the development of PDA in Kras^G12D^-expressing mice models (Aguirre et al. 2003; Hingorani et al. 2005). Remarkably, we did not observe mutations in *p53*, *p16*, and *Smad4* in the tumors derived from ATDC-overexpressing mice, and this was supported by functional assays (Supplemental Fig. 3; Supplemental Table 1). This suggests that physiologic ATDC overexpression (similar to that seen in human tumors) is sufficient to accelerate Kras-mediated tumorigenesis and enhance metastasis in the absence of common alterations in tumor suppressors in PDAC.

A central finding of our study is that ATDC promotes a metastatic phenotype with EMT cellular features. During malignant progression, it has been proposed that carcinoma cells undergo an EMT, a developmental process that allows cells that are part of a rigid epithelial architecture to delaminate and translocate (Valastyan and Weinberg 2011; Tuveson and Neoptolemos 2012). Most studies of EMT related to cancer have been conducted in vitro; thus, the relevance of EMT to carcinogenesis continues to be debated. Our AKC TG mouse model provides some insight into the in vivo role of EMT, and our data showed that the EMT markers Snail1 and Zeb1 are highly up-regulated in PanIN lesions and PDA samples from AKC and A^LGSL^KC mice. This suggests that ATDC is an inducer of EMT and that this may be an important component of the oncogenic functions of ATDC.

In human pancreatic cancer, vimentin and fibronectin are up-regulated in high-grade tumors and within poorly differentiated areas of low-grade tumors, with a concurrent decrease in E-cadherin expression. These markers of EMT are often associated with poor patient outcomes. In resected PDA specimens, nearly 80% demonstrated moderate to strong SNAIL1 expression, while only 50% showed SLUG expression, with very few revealing elevated TWIST expression (Hotz et al. 2007). SNAIL1 expression was inversely correlated with E-cadherin expression, with decreased E-cadherin expression associated with higher tumor grade (Hotz et al. 2007). ZEB1 expression in pathologic specimens correlated with advanced tumor grade and worse outcomes and had an inverse relationship with E-cadherin (Buck et al. 2007; Arumugam et al. 2009). To determine whether ATDC overexpression exclusively accelerates tumor formation or also affects the histology of tumors that form, we carefully examined the PDA samples from AKC and A^LGSL^KC mice and quantified our results in a manner similar to that done with KPC mice by Hingorani et al. (2005) (Supplemental Table 2). Since only three PDAs were generated from 30 KC mice in our study, these numbers were really inadequate for a direct comparison. In the KPC model description (Hingorani et al. 2005), the majority of KPC mice (22 out of 25) generated PDAs with glandular architecture, and only a small portion of those PDAs contained undifferentiated (seven out of 25) or sarcomatoid (two out of 25) regions. Our analysis showed that eight out of 30 AKC or four out of 10 A^LGSL^KC mice exhibited sarcomatoid regions of PDA, and seven out of 30 AKC or three out of 10 A^LGSL^KC mice had undifferentiated regions of PDA. The frequency of sarcomatoid or undifferentiated features was higher in PDA from AKC or A^LGSL^KC mice than reported with KPC mice. However, in the absence of a direct comparison of AKC and KPC tumors, we conclude that ATDC overexpression accelerates tumor formation and may induce the formation of tumors less differentiated than those observed in KPC mice.

In this study, we found that the EMT markers Snail1 and Zeb1 were significantly increased in PanIN lesions and PDA in AKC and A^LGSL^KC mice. Furthermore, we found a strong linkage between ATDC overexpression, EMT, and tumor invasion and found that ATDC colocalized with Snail1 or Zeb1 in not only AKC and A^LGSL^KC mice but also early PanIN lesions from KC mice and human pancreatic tissues. Using the KPCY mouse model, which allows lineage tracing of pancreatic epithelial cells, we observed that ATDC- and YFP-double-positive epithelial cells migrated into the stroma surrounding PanIN lesions. These ATDC-positive cells coexpressed Zeb1 or Snail1, suggesting that these cells have undergone EMT. Finally, knockdown of ATDC in primary PDA cell lines derived from AKC mice significantly decreased Snail1 and Zeb1 gene expression and prohibited invasion. Based on these observations, we propose that ATDC is a critical regulator of premalignant pancreatic epithelial cell dissemination in PanIN stage lesions by promoting EMT.

Aggressive metastatic dissemination and resistance to conventional treatment options remain the greatest hurdles in treating PDA despite an increasing understanding of the genetic makeup of the disease (Biankin et al. 2012; Vogelstein et al. 2013). Here we delineated a novel molecular pathway for EMT in pancreatic tumorigenesis, showing that ATDC is a proximal regulator of EMT. This study further substantiates the important role of ATDC in PDA development and sheds new light on strategies for treatment, indicating that ATDC is a potential target of therapeutic intervention in PDA.

## Materials and methods

### Animal models

To generate TG mice expressing the ATDC transgene, Flag-tagged ATDC cDNA (provided by Dr. J. Murnane, University of California at San Francisco) was cloned into the pCAGGS vector (purchased from Belgium Coordinated Collections of Micro-organisms [BCCM]/LMBP, Ghent University, Gent-Zwijnaarde, Belgium) containing a CMV immediate early enhancer, a chicken β-actin gene promoter, and a rabbit β-globin splice acceptor (CAG promoter) (Kanegae et al. 1995), a promoter/enhancer that drives strong gene expression in most tissues. To generate pancreas-specific expression of the ATDC transgene, we used a pCLE vector containing a CAG promoter and a floxed GFP/stop cassette. Flag-tagged ATDC was subcloned into the pCLE vector after the floxed GFP/stop cassette (ATDC^LGSL^). The CAG-ATDC and ATDC^LGSL^ plasmids were linearized with restriction enzyme digestion, and CAG-ATDC and ATDC^LGSL^ TG mice were produced by microinjecting the linearized plasmids into the pronuclei of the fertilized eggs from the FVB/JN mice (Jackson Laboratory) at the University of Michigan Transgenic Animal Model Core. CAG-ATDC and ATDC^LGSL^ TG mice were identified by PCR assays of genomic DNA described in the Supplemental Material. LSL-Kras^G12D^, p48-Cre, and Trp53^R172H^ mice and p53^f/+^; LSL-Kras^G12D^;Pdx1-Cre;RosaYFP mice have been described previously (Rhim et al. 2012). All animal care and procedures were approved by the Institutional Animal Care and Use Committee at the University of Michigan.

### Constructs

The Myc-tagged expression vectors of human and mouse TCF4, β-catenin, CD44 (transcript variant 1), Snail1, and Zeb1 were purchased from OriGene. Lentiviral constructs expressing control and shRNA targeting human and mouse ATDC, β-catenin, CD44, Snail1, and Zeb1 were purchased from Santa Cruz Biotechnology.

### Mutation analysis of the Kras, p53, Cdkn2/Ink4a, Smad4, and Cdk4 genes

Pancreatic tumor samples or cell lines were prepared as previously described for mutational analysis (Hingorani et al. 2005). In brief, RNA extraction from cells was performed using the RNeasy minikit (Qiagen), and cDNA was prepared using the first strand cDNA synthesis kit (GE Healthcare Biosciences). PCR using Ampli Taq polymerase (Life Technologies) mixed with PfuUltra HF DNA polymerase (Stratagene) was performed to amplify the Kras, Trp53, Cdkn2a/p16Ink4a, Dpc4/Smad4, and Cdk4 genes using PCR primer pairs as described previously (Hingorani et al. 2005). PCR products were purified using the PureLink PCR purification kit (Life Technologies) and sequenced by the University of Michigan DNA Sequencing Core.

### CD44 luciferase reporter gene assays

The CD44 reporter gene construct was purchased from GeneCopoeia, Inc. Luciferase assays were performed using a Secrete-Pair dual-luminescence assay kit (GeneCopoeia, Inc.). A secondary reporter gene, secreted alkaline phosphatase (SEAP), was used for transfection normalization. The Gaussia luciferase activity was normalized by following formula: relative light units (RLU) = [sample Gaussia RLU/(sample SEAP RLU/average SEAP RLU)].

### Quantitative ChIP assays

ChIP analysis for TCF4 occupancy on the promoter region of the CD44 gene was performed using the SimpleChIP Plus enzymatic ChIP kit (Cell Signaling). Briefly, formaldehyde cross-linked chromatin was isolated from 5 × 10^7^ pancreatic cancer cells derived from AKC mice and sonicated into 500- to 1000-bp fragments. Ten micrograms of chromatin samples was diluted with ChIP dilution buffer to a final volume of 1 mL. Two percent of the sample was used as input chromatin. The remaining chromatin was incubated with 2 μg of ChIP-grade TCF4 antibody (Cell Signaling) and normal rabbit IgG (Santa Cruz Biotechnology) overnight at 4°C. ChIP-grade magnetic beads (Cell Signaling) were used for the pull-down. ChIP-enriched DNA was quantified by quantitative real-time PCR using primer sets described in Supplemental Table 4. The quality of the ChIP procedure was confirmed using PCR primers corresponding to a well-known β-catenin target sequence in the Axin2 promoter as a positive control and a region of the GAPDH coding region as a negative control.

Immunoprecipitated chromatin was normalized to input chromatin, and data are expressed as percentage of input.

### Statistical analysis

Statistical analysis was performed using the StatMate software package (GraphPad Software, Inc.) and two-way analysis of variance (ANOVA). Results were expressed as the mean ± standard deviation. In cases of statistically significant differences, a Student’s *t*-test was used to determine which groups statistically differed from each other. Statistical significance was accepted if *P* < 0.05.

## Supplementary Material

Supplemental Material
