# Supplementary material for: ATDC induces an invasive switch in KRAS-induced pancreatic tumorigenesis
Source: Genes Dev. 2015 Jan 15;29(2):171–83. doi: 10.1101/gad.253591.114 (PMC4298136; doi:10.1101/gad.253591.114)
Supplement: Supplemental Material [file supp_29_2_171__index.html]

Supplemental Material 

# ATDC induces an invasive switch in KRAS-induced pancreatic tumorigenesis

## Supplemental Material

**Files in this Data Supplement:**

- Supp Figures & Tables.pdf
- Supp Methods & Legends.pdf
