# Supplementary material for: ATDC induces an invasive switch in KRAS-induced pancreatic tumorigenesis
Source: Genes Dev. 2015 Jan 15;29(2):171–83. doi: 10.1101/gad.253591.114 (PMC4298136; doi:10.1101/gad.253591.114)
Supplement: Supplemental Material [file supp_29.2.171_SuppFigs1-17_Tables.pdf]

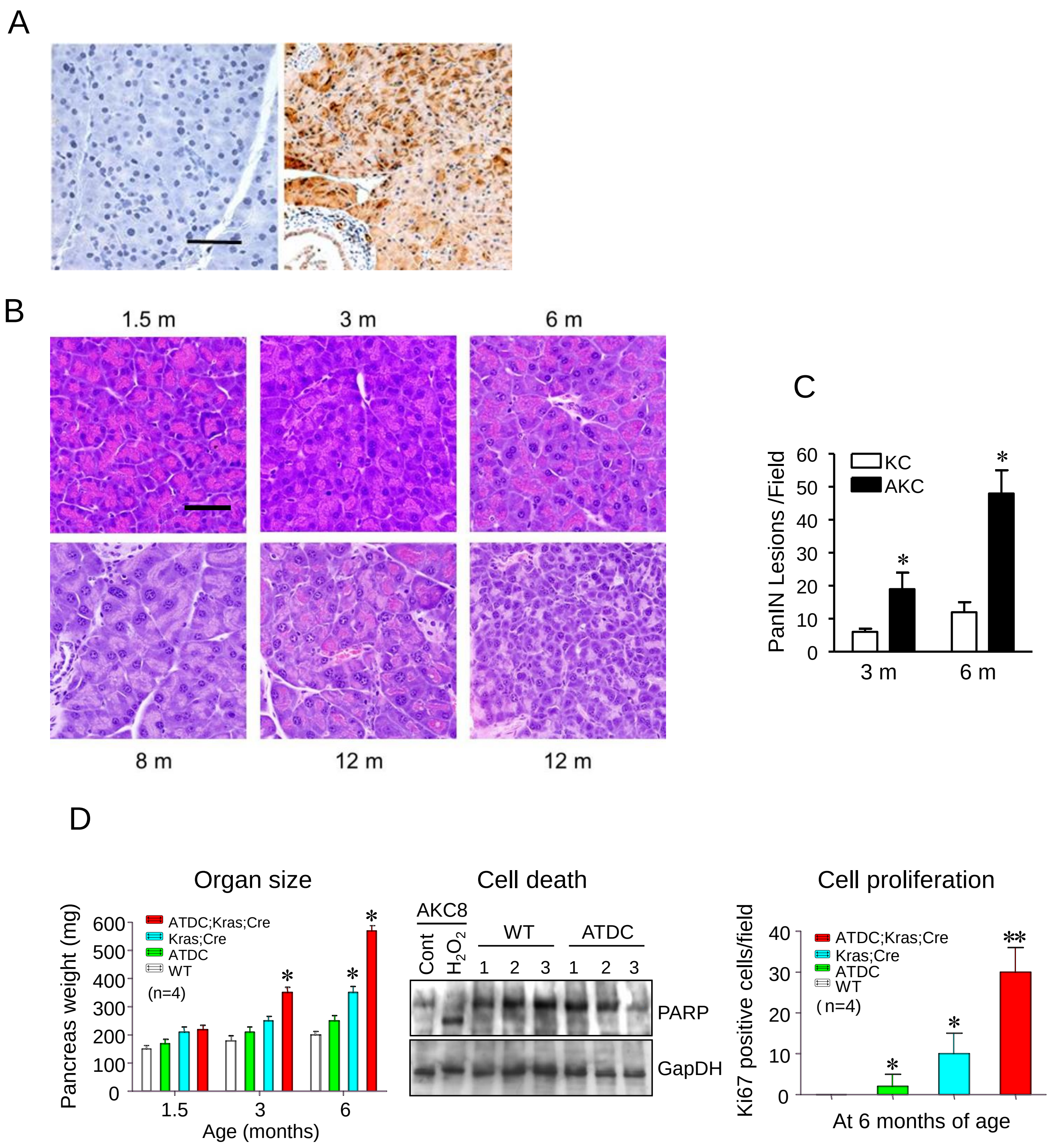

Supplemental Figure 1

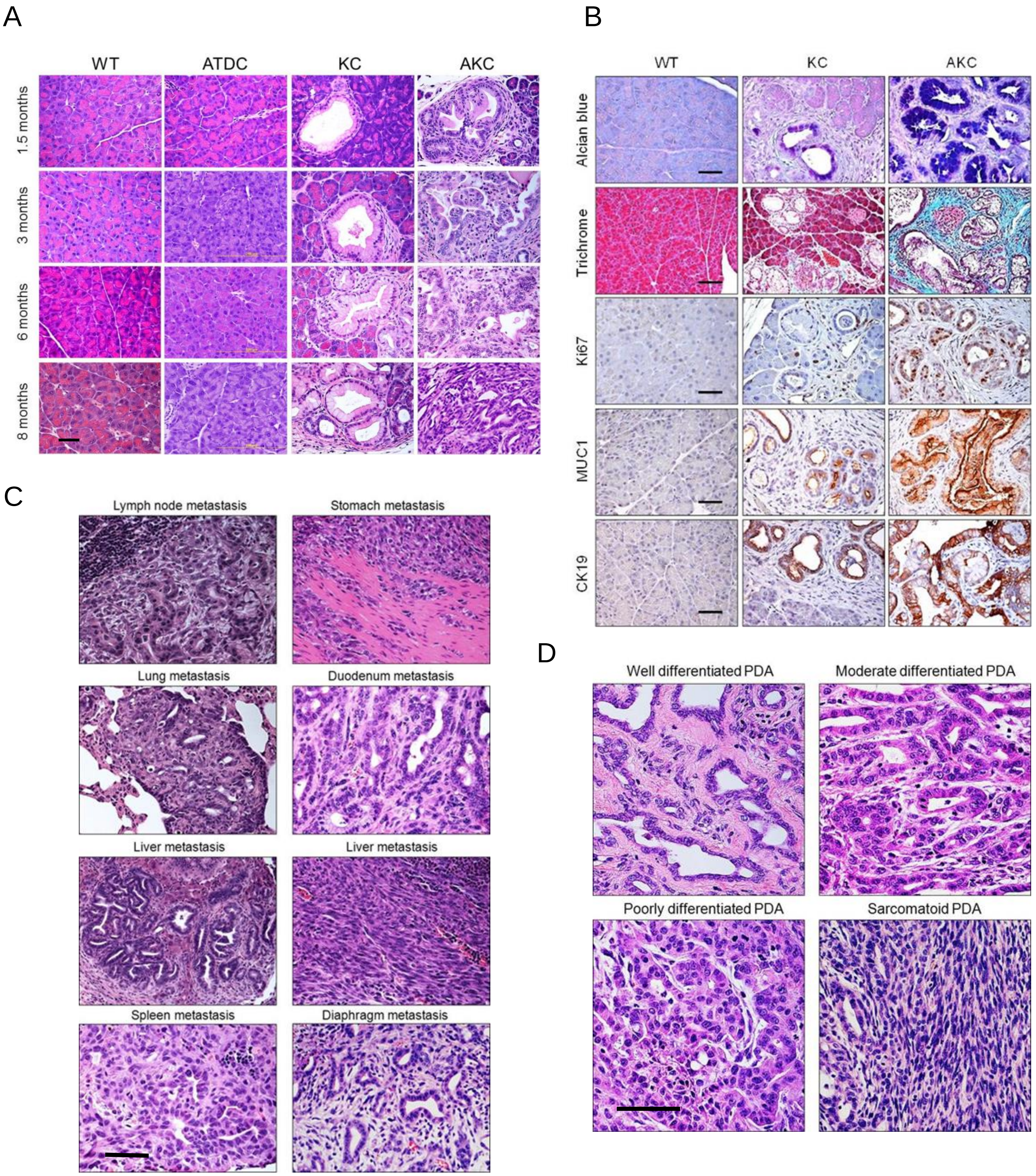

Supplemental Figure 2

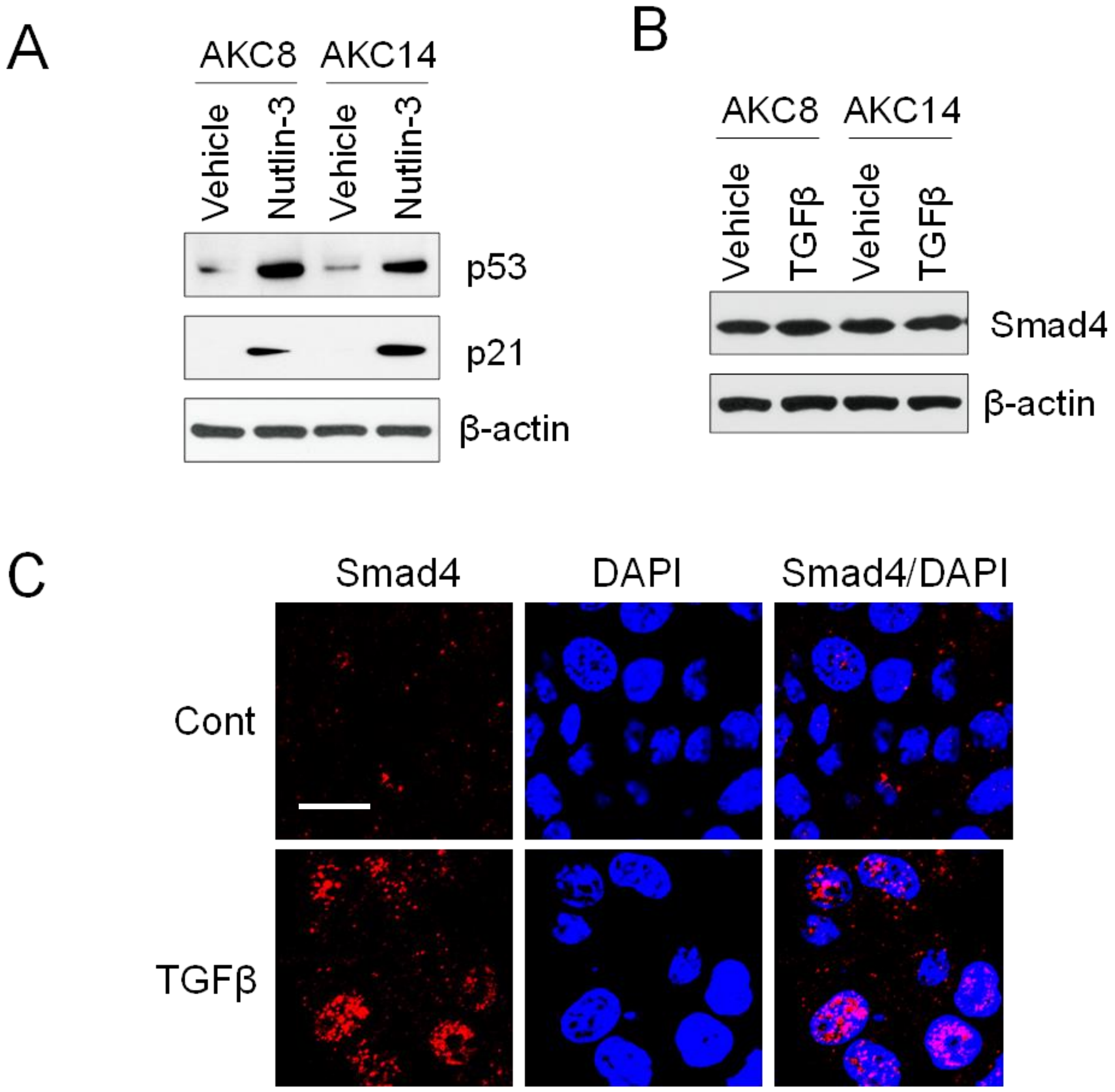

Supplemental Figure 3.

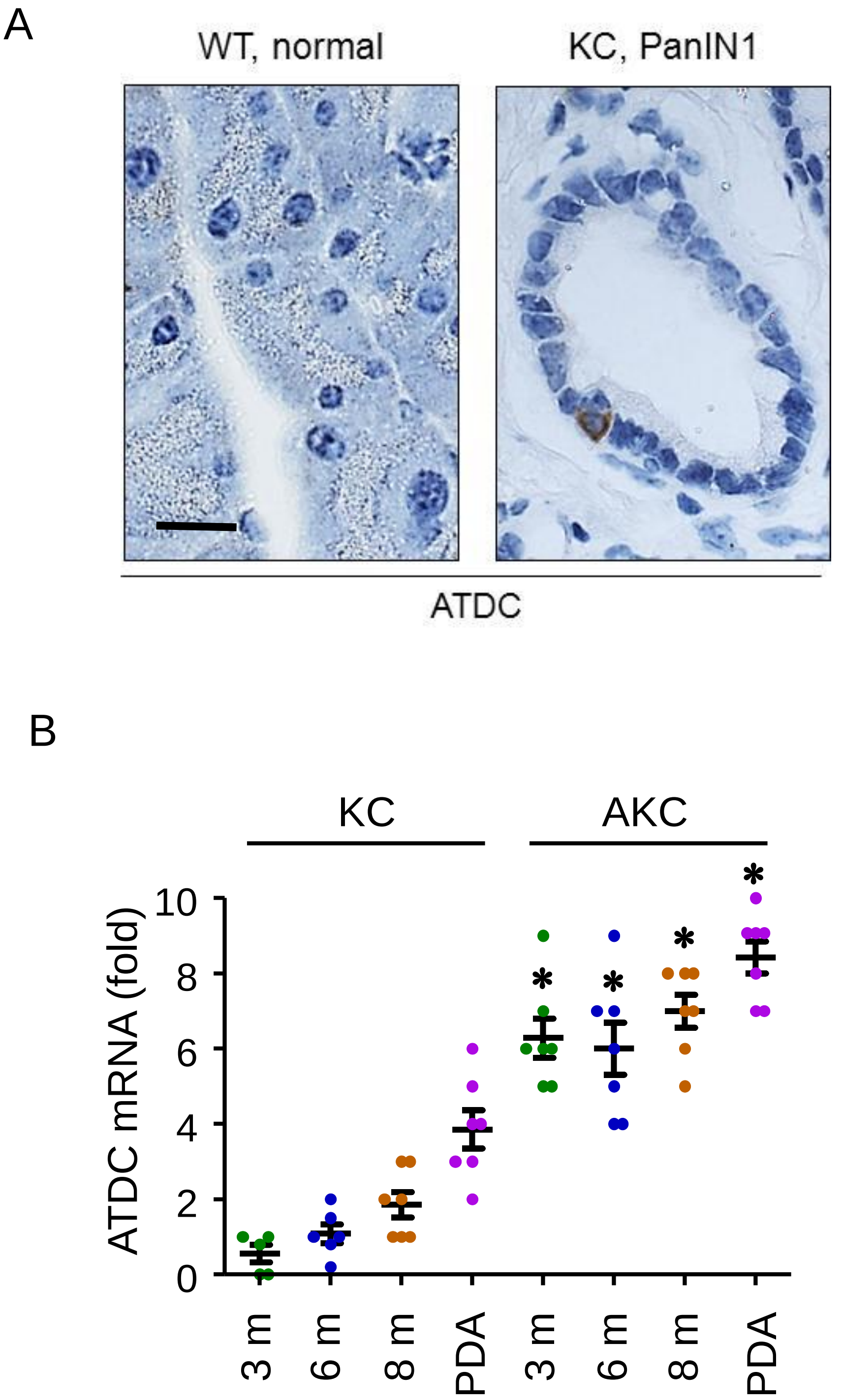

Supplemental Figure 4.

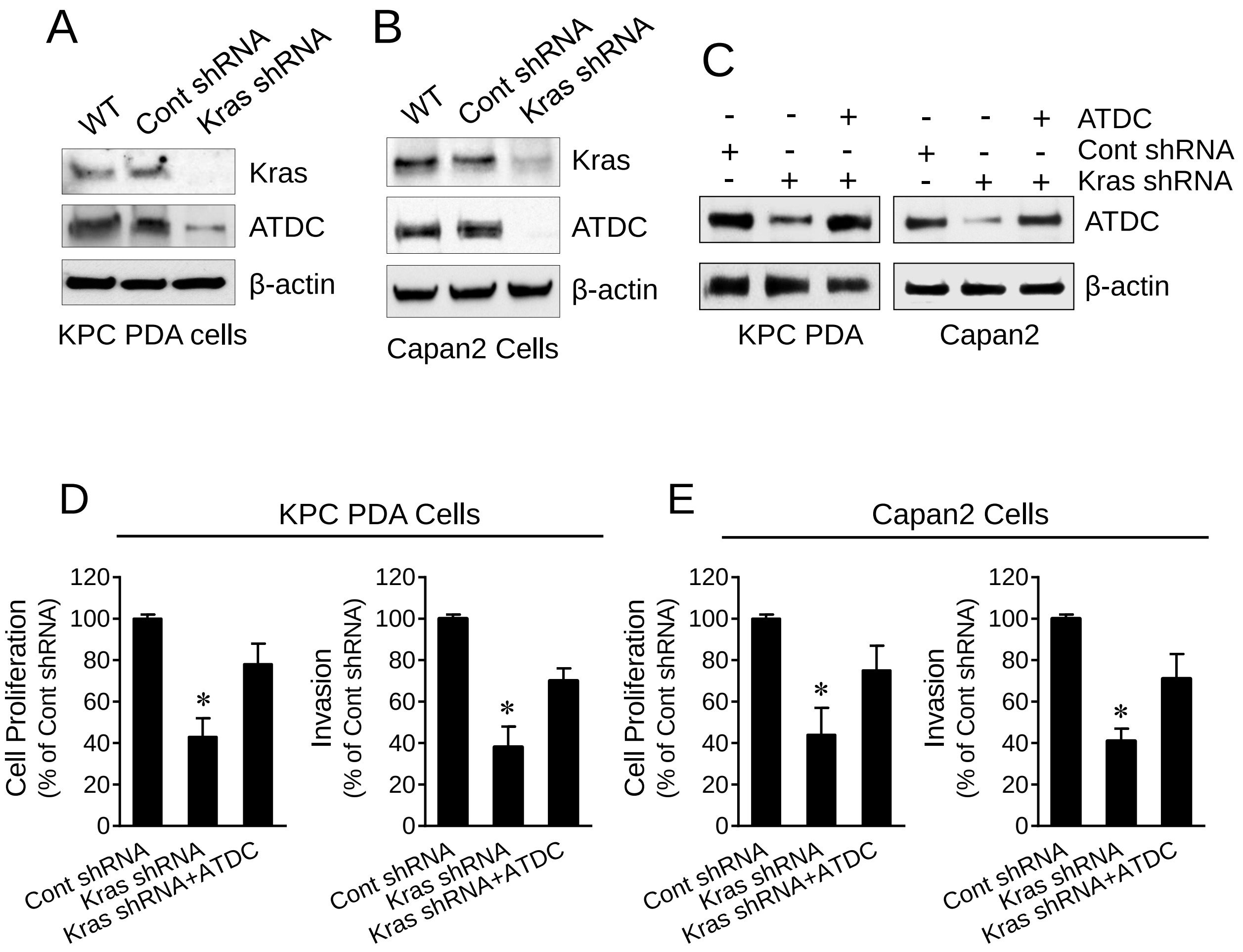

Supplemental Figure 5

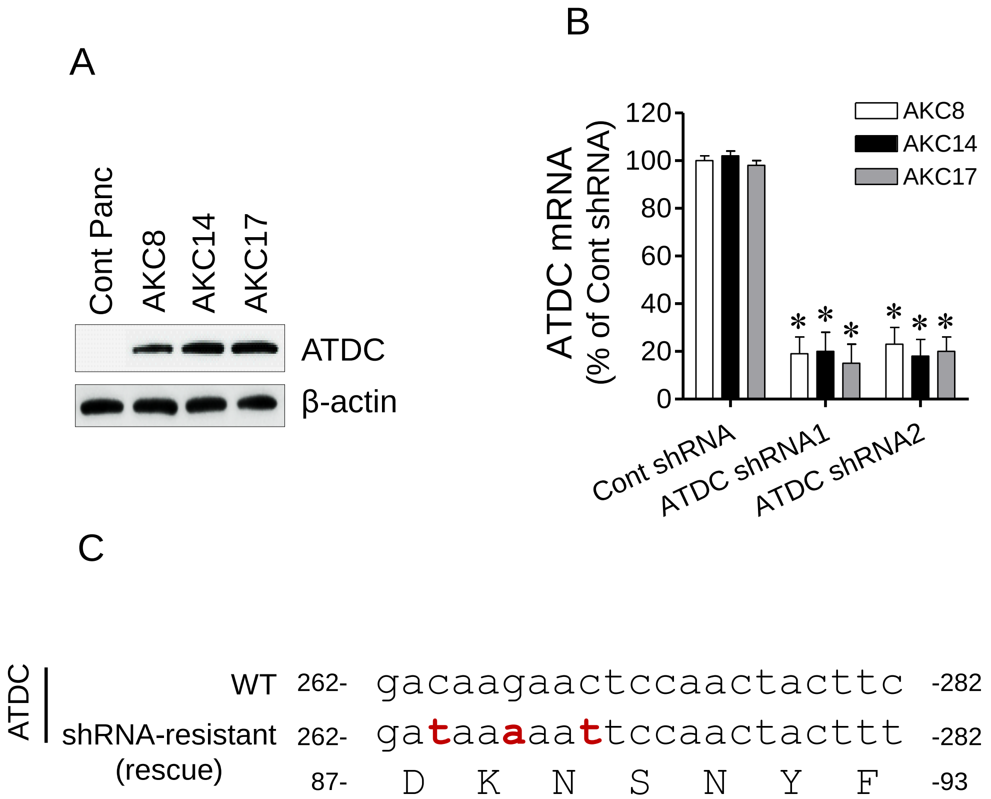

Supplemental Figure 6.

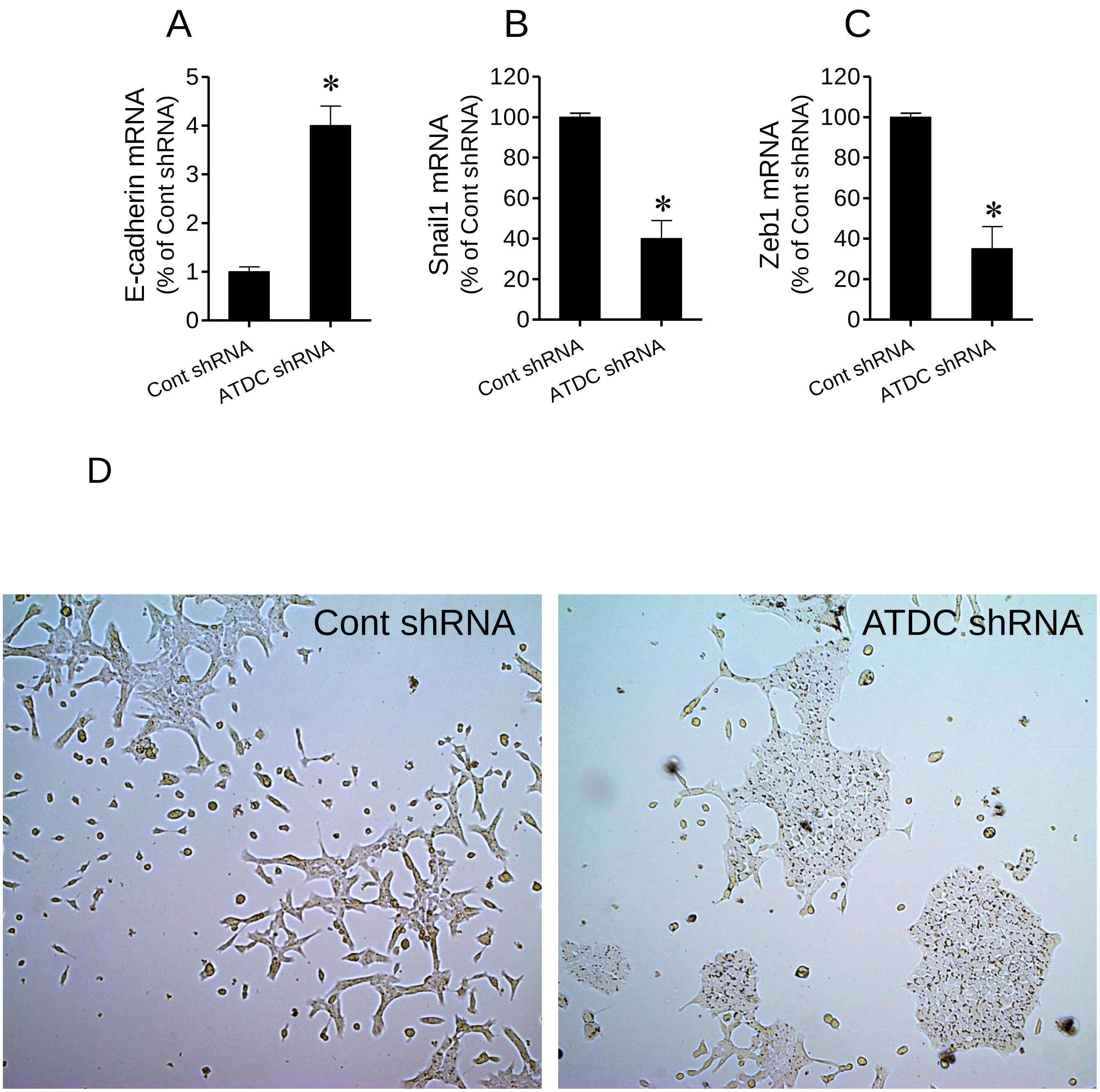

Supplemental Figure 7

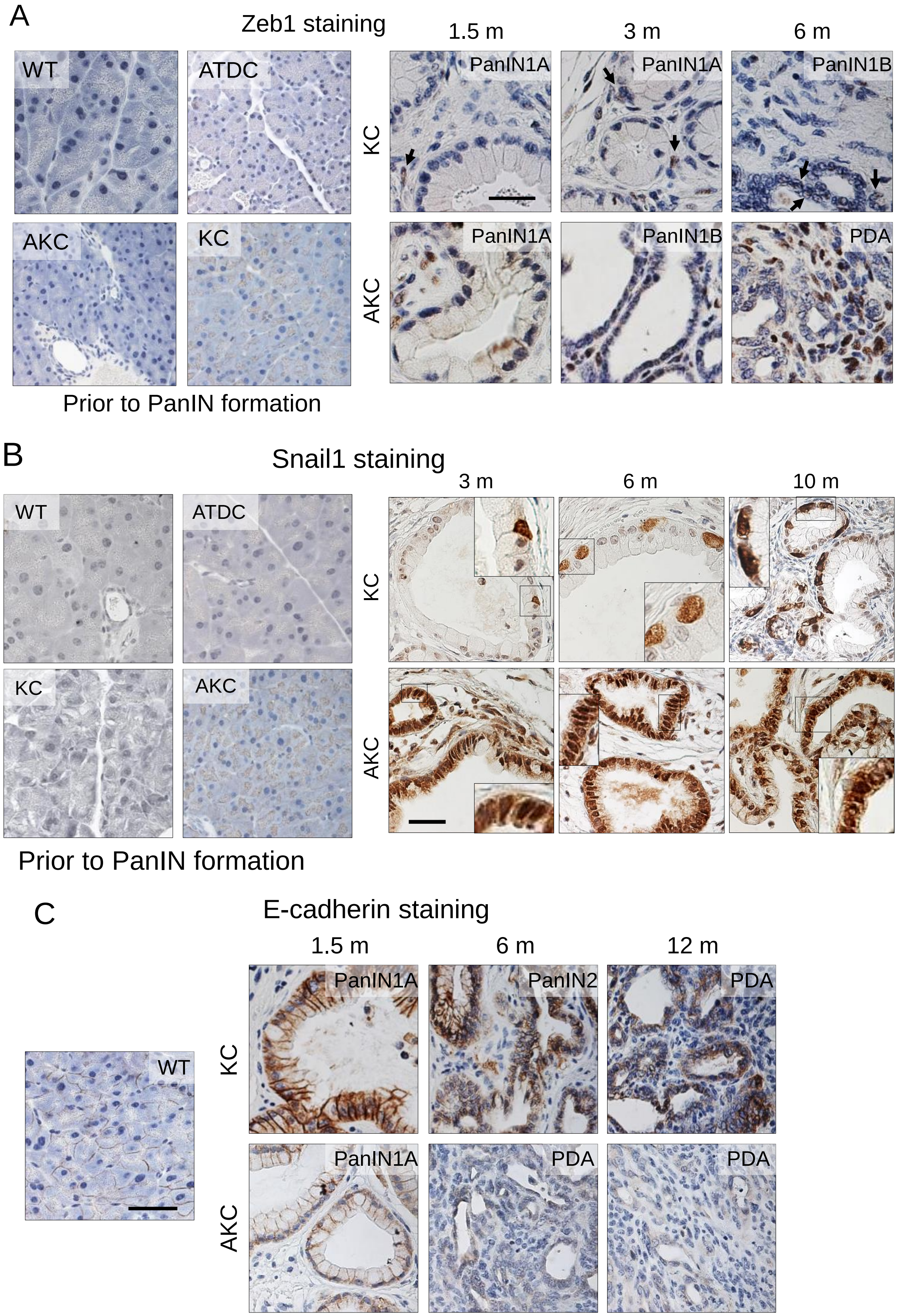

Supplemental Figure 8

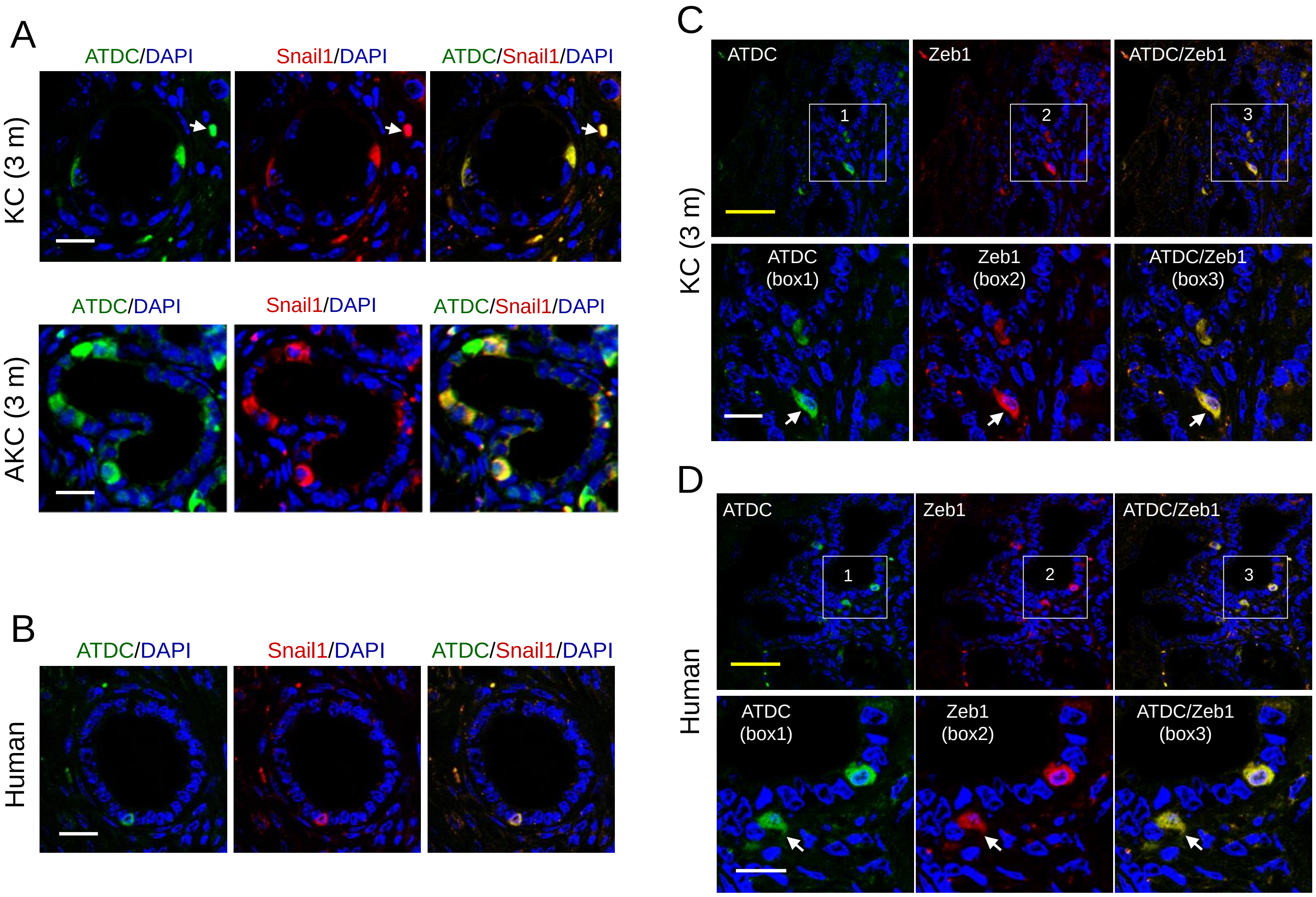

Supplemental Figure 9.

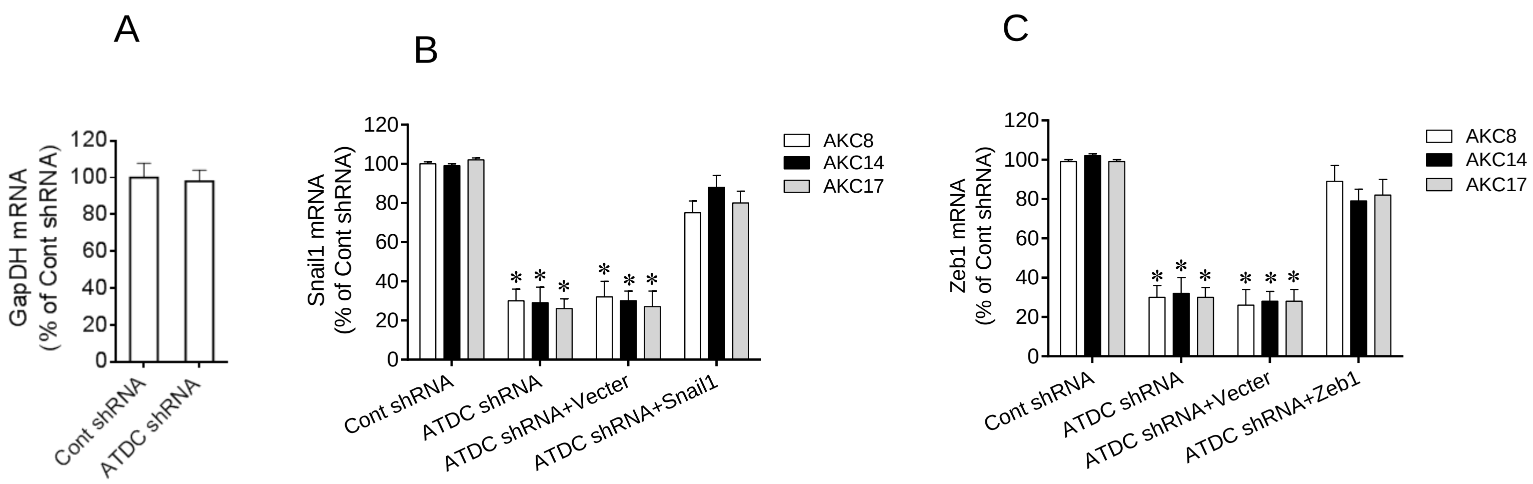

Supplemental Figure 10

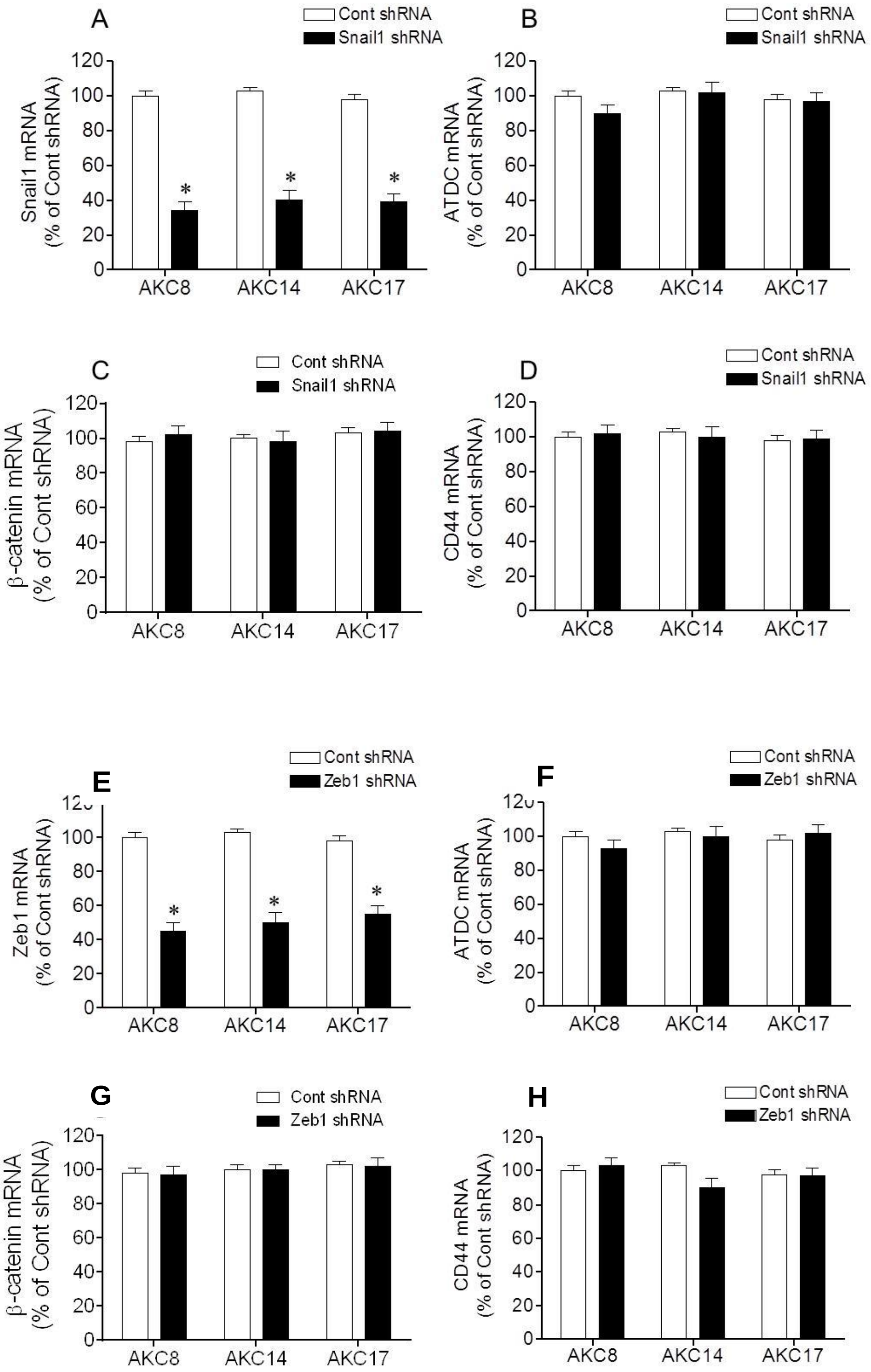

Supplemental Figure 11

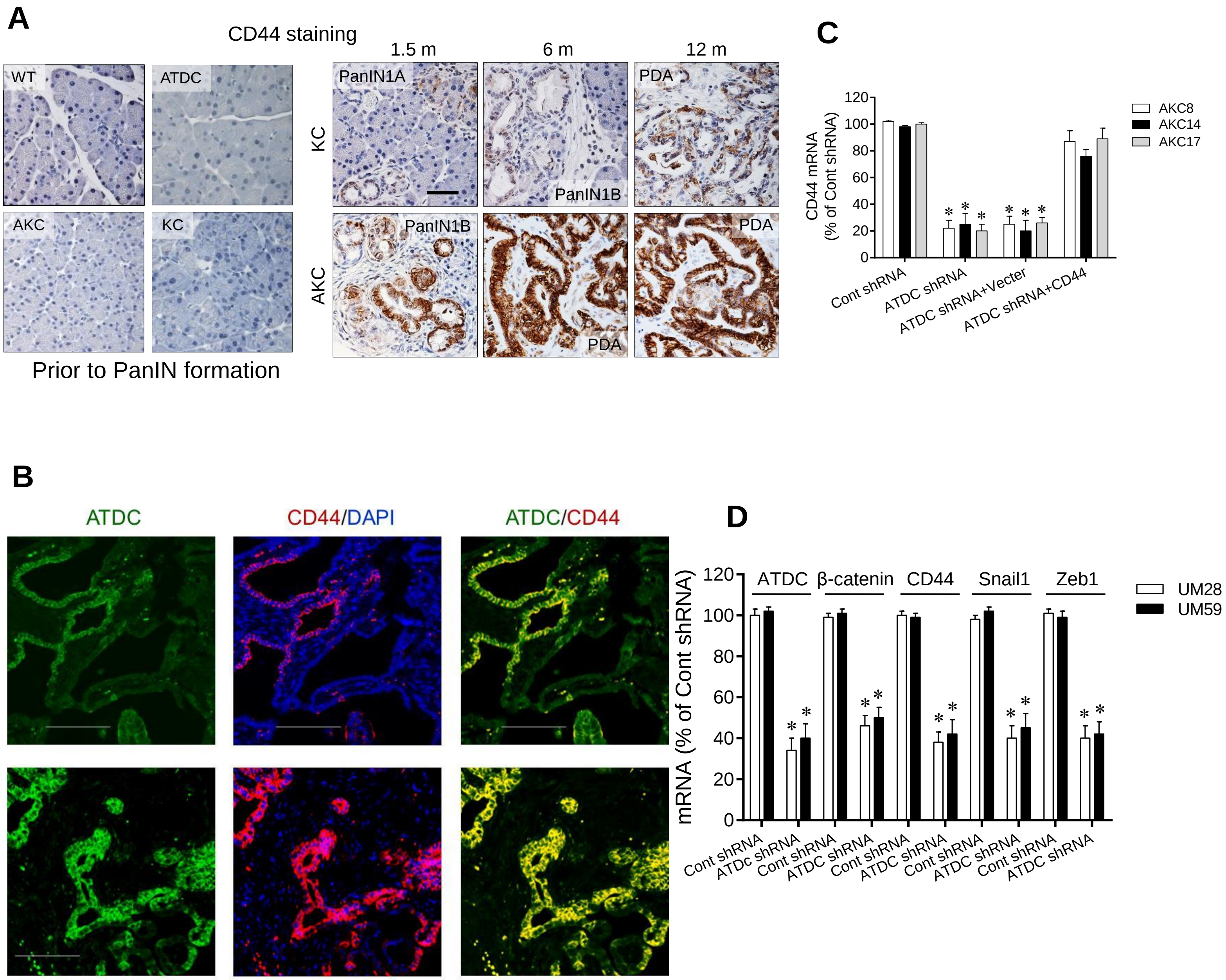

Supplemental Figure 12

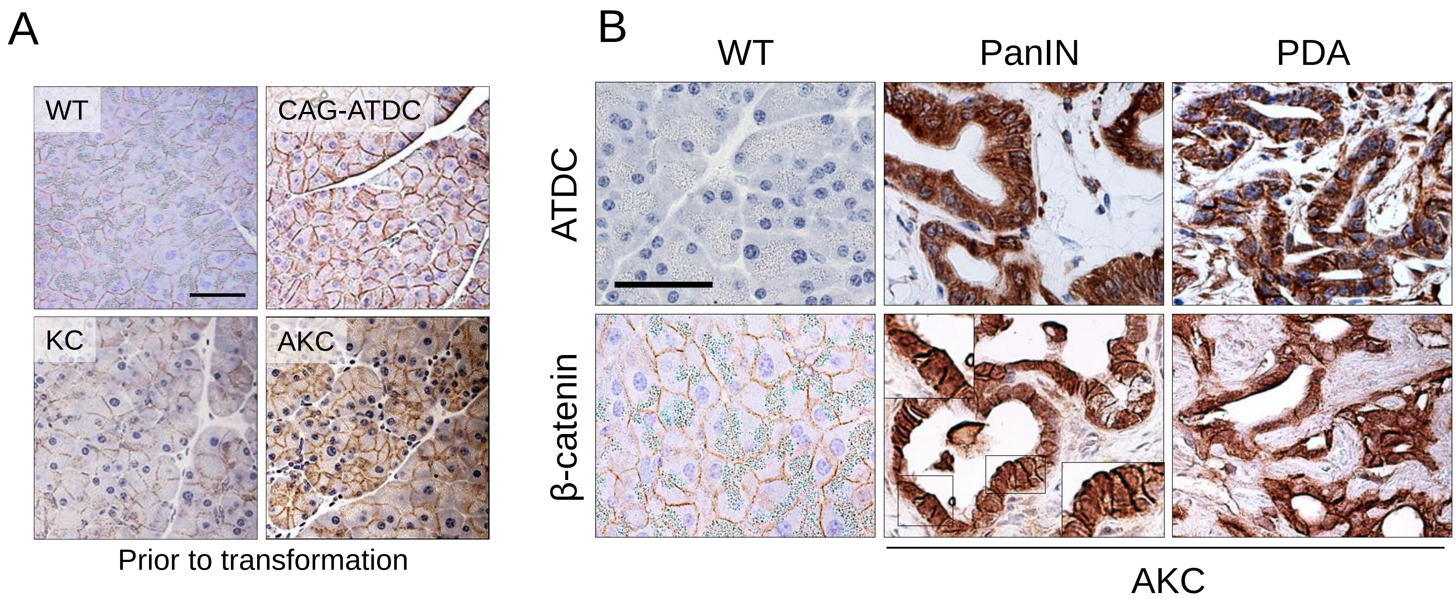

Supplemental Figure 13

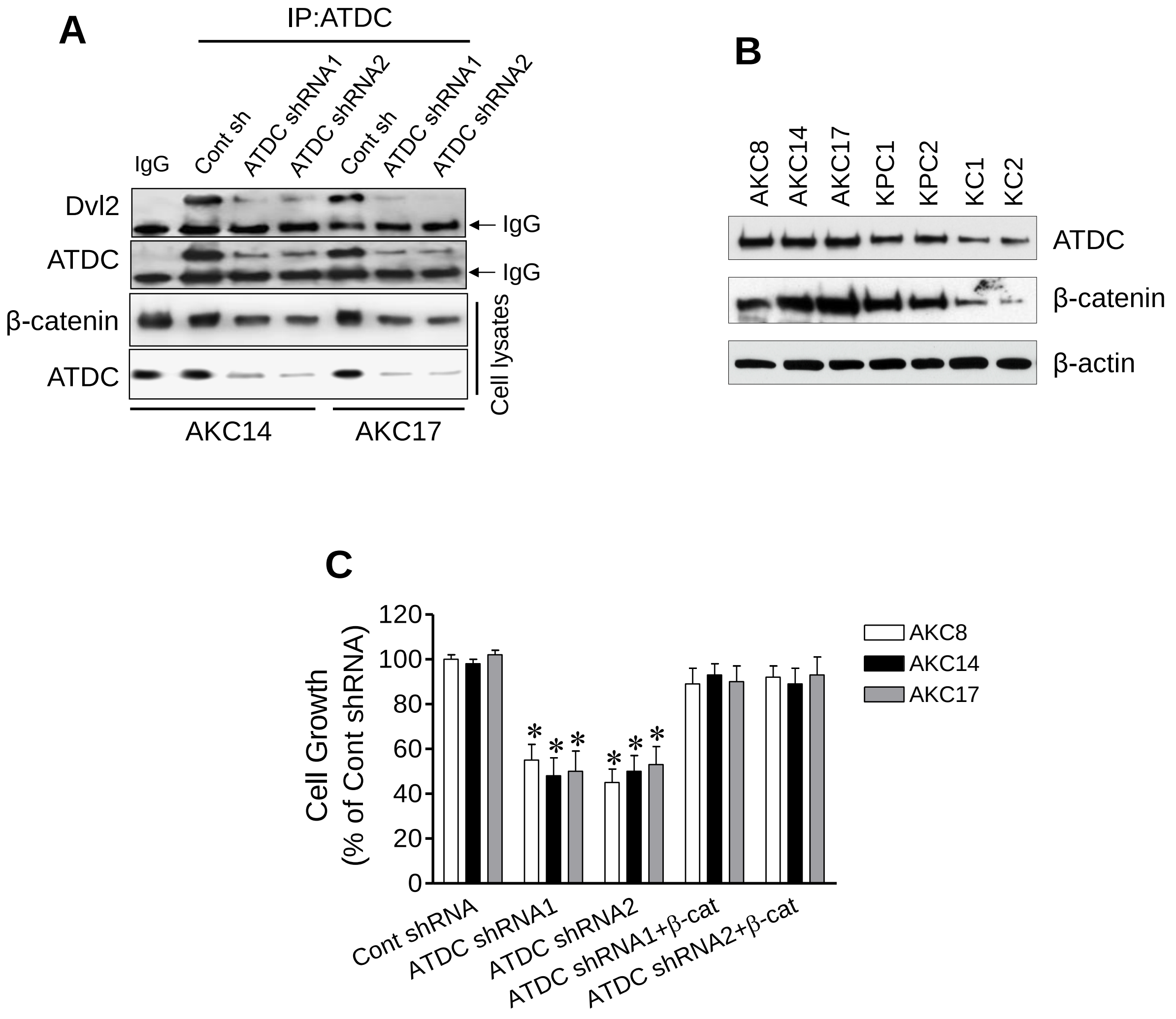

Supplemental Figure 14

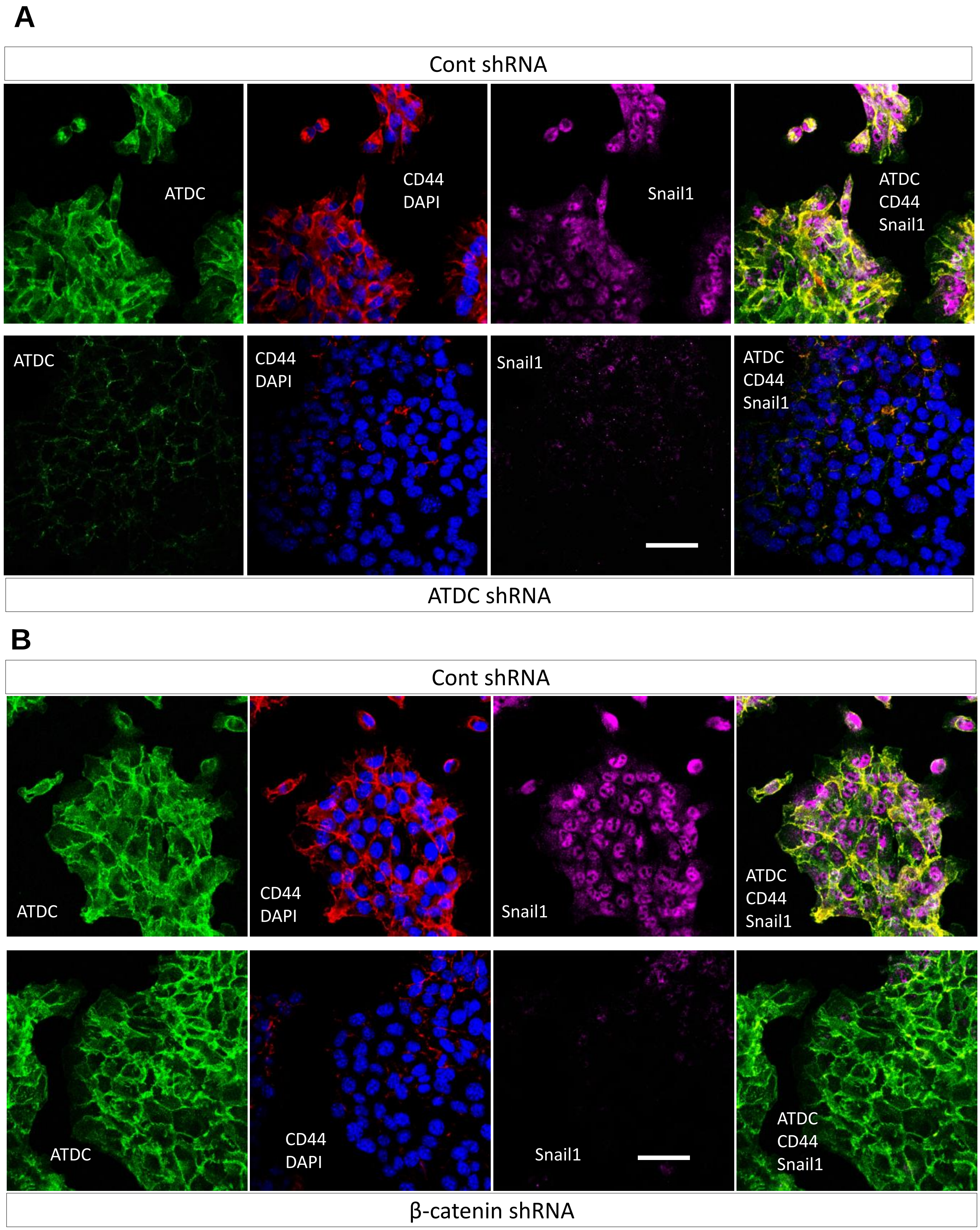

Supplemental Figure 15

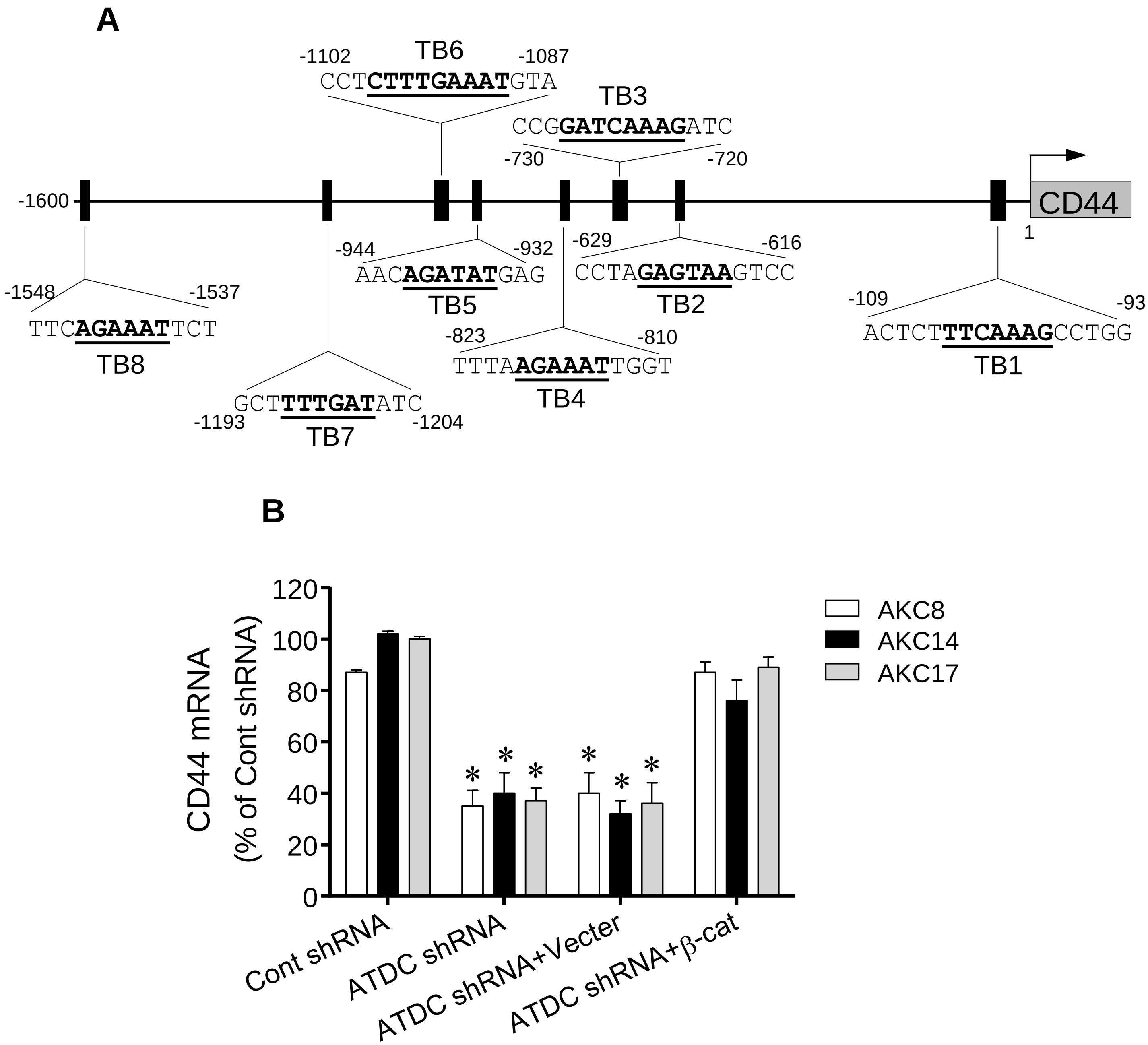

Supplemental Figure 16

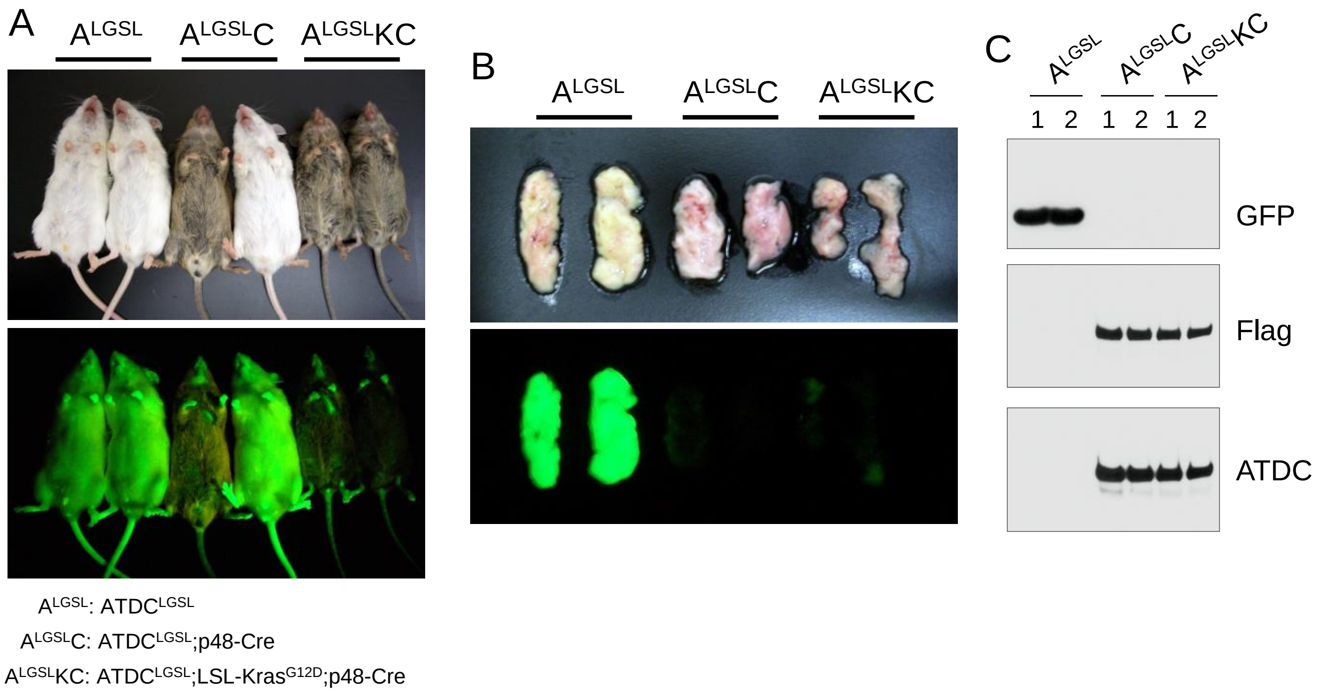

Supplemental Figure 17

Supplemental Table 1. Genetic Profile of PDA From *ATDC;Kras^{G12D};p48-Cre* (AKC) Mice

PDA (AKC)	ATDC	Kras	p53	P16^{Ink4}	Smad4	CDK4
AKC5	Overexpression	Mut (G12D)	WT	WT	WT	WT
AKC6	Overexpression	Mut (G12D)	WT	WT	WT	WT
AKC7	Overexpression	Mut (G12D)	WT	WT	WT	WT
AKC9	Overexpression	Mut (G12D)	WT	WT	WT	WT
AKC11	Overexpression	Mut (G12D)	WT	WT	WT	WT
AKC13	Overexpression	Mut (G12D)	WT	WT	WT	WT
AKC19	Overexpression	Mut (G12D)	WT	WT	WT	WT
AKC23	Overexpression	Mut (G12D)	WT	WT	WT	WT
PDA cell lines (AKC)						
AKC8	Overexpression	Mut (G12D)	WT	WT	WT	WT
AKC14	Overexpression	Mut (G12D)	WT	WT	WT	WT
AKC17	Overexpression	Mut (G12D)	WT	WT	WT	WT
PDA cell lines (PKC)	N/A	Mut (G12D)	Mut (R172H)	WT	WT	WT
PDA cell lines (Ink4KC)	N/A	Mut (G12D)	WT	Deletion	WT	WT

PKC: Trp53^{R172H};Kras^{G12D};p48-Cre mice.
Ink4KC: Ink4a/Ar$^{flox/lox}$;Kras^{G12D};p48-Cre mice

Supplemental Table 2. Histology of PDA in AKC and $A^{LGSL}KC$ mice

AKC				$A^{LGSL}KC$		
	PDA Histology				PDA Histology	
	1°	2°			1°	2°
1	S			1	G	
2	S	G		2	G	U; S
3	G	U; S		3	S	G
4	G			4	G	
5	G			5	G	S
6	G	U		6	G	U
7	G			7	G	
8	G			8	G	U
9	G			9	G	S
10	G	U; S		10	G	
11	G					
12	G					
13	G					
14	G	U				
15	G					
16	G					
17	G	U				
18	G	S				
19	S	G				
20	G					
21	G					
22	G	U;S				
23	G					
24	G					
25	G					
26	G	U;S				
27	G					
28	G					
29	G					
30	G					

1°, predominant pancreatic histology noted (present in >50% of evaluated tissue); 2°, secondary pancreatic histologies noted; G, glandular; U, undifferentiated; S, sarcomatoid.

Supplemental Table 3. Antibodies

Antibody	Supplier	Catalog number	IHC dilution	IF dilution	WB dilution
Beta-Catenin	Cell Signaling	9587	1:100	-	-
CK19 (TimaIII)	Iowa Developmental Hybridoma Bank	-	1:100	1:100	
E-Cadherin	BD	610182	1:200	1:200	-
Ki67	Vector Laboratories	VP-RM04	1:100		
MUC1	Thermo Scientific	HM1630-P	1:100	-	-
p-ERK1/2	Cell Signaling	4370	1:100	-	1:1000
GFP	Abcam	ab5450	1:500	1:500	-
Zeb1	Santa Cruz	sc-25388	1:200	1:200	-
Snail1	Abcam	ab53519	1:100	1:100	-
CD44	Abcam	ab119863	1:200	1:200	-
ATDC	Sigma	HPA020053	1:200	1:200	-
ATDC	Santa Cruz	sc-166707	-	-	1:1000
Beta-catenin	Cell Signaling	8480	1:200	1:200	1:1000
Smad4	Santa Cruz	sc-7966	-	1:200	1:1000
Alexa Fluor® 488 Donkey Anti-Rabbit	Invitrogen	A-21026	-	1:400	-
Alexa Fluor® 568 Donkey Anti-Mouse	Invitrogen	A-10037	-	1:400	-
Alexa Fluor® 568 Donkey Anti-Rat	Invitrogen	A-10042	-	1:400	-
Alexa Fluor® 568 Donkey Anti-Goat	Invitrogen	A-11057	-	1:400	-
Alexa Fluor® 647 Donkey Anti-Goat	Invitrogen	A-21447	-	1:400	-

Supplemental Table 4: Quantitative real-time PCR primers for ChIP assay

	Forward	Reverse
TB1	ggctgtgtacatttctctcactttctc	gctgaatgagaaacacagaaggc
TB2	gccatgccttctgtgtttc	attgcaattccctatcttctctaa
TB3	ttagagaagatagggaattgcaat	attcccctttacatcttgatacatttc
TB4	tgagtggatatggggatgggtaga	ctgcacaacttttcaatccttttc
TB5	gacagaggaatggatgggtgg	caacacatttcattcatgctctca
TB6	tgagagcatgaatgaaatgtgttg	gtggggactgaaaagtgggg
TB7	gatctctccctctccctctctctc	ctgggacaaggagaatgactgag
TB8	cttccgttggctgcttagtc	ggtttttattccaggctttga

Supplemental Table 5: TaqMan primers for quantitative real-time PCR primers

Mouse		Human	
Zeb1	Mm00495564_m1	Zeb1	Hs00232783_m1
Snail1	Mm00441533_g1	Snail1	Hs00195591_m1
β-catenin	Mm00483039_m1	β-catenin	Hs00355049_m1
CD44	Mm01277163_m1	CD44	Hs01075861_m1
ATDC	Mm01175102_m1	ATDC	Hs00232590_m1
E-cadherin	Mm01247357_m1	E-cadherin	Hs01023894_m1
Slug	Mm00441531_m1	Slug	Hs00950344_m1
GapDH	Mm99999915_g1	GapDH	Hs99999905_m1

Supplemental Table 6. Quantification of double positive Zeb1 or Snail1/ATDC cells in PanIN mice. Numbers reflect evaluation of ten medium-powered fields from each of five PanIN mice.

Grade	KC		AKC	
	Zeb1 or Snail1/ATDC double positive cells	*Epithelial PanIN cells*	*Zeb1 or Snail1/ATDC double positive cells*	*Epithelial PanIN cells*
**PanIN1**	9 (1.8%)	476	19 (5.9%)	319
**PanIN2**	39 (7.3%)	532	62 (14.5%)	428
**PanIN3**	47 (14.5%)	324	98 (28.7%)	341
