# Supplementary material for: ATDC induces an invasive switch in KRAS-induced pancreatic tumorigenesis
Source: Genes Dev. 2015 Jan 15;29(2):171–83. doi: 10.1101/gad.253591.114 (PMC4298136; doi:10.1101/gad.253591.114)
Supplement: Supplemental Material [file supp_29.2.171_SuppMeth_FigLegends.pdf]

SUPPLEMENTAL MATERIALS
Part one: Supplemental Methods

Quantitative RT-PCR

RNA extraction and reverse transcription was performed using a RNeasy mini kit (QIAGEN, Valencia, CA) and a High-Capacity cDNA Reverse Transcription Kit (Life Technologies, Grand Island, NY), respectively. Samples were prepared using the SYBR Green PCR Master Mix (Life Technologies, Grand Island, NY). Primer sequences and reaction conditions are described in the Supplemental table 5.

Immunoblot Analysis

Western blot analysis and co-Immunoprecipitation (co-IP) was done as previously described (1). The following primary antibodies were used (Supplemental table 3). After incubation with the secondary HRP-conjugated anti-rabbit (1:5000) antibody, protein bands were visualized on Kodak Biomax XAR film. After analysis, blots were stripped, washed and reprobed with a β-actin antibody (Sigma, St Louis, MO) which served as a loading control. Protein expression was quantified using a Kodak Gel Documentation System (model 1D 3.6).

Histology, Immunohistochemical and Immunofluoresence Analysis

Pancreatic tissues were fixed overnight in 10% neutral buffered formalin and were embedded in paraffin, and sectioned by the University of Michigan Cancer Center Histopathology Core. H&E, PAS, Gomori trichrome, and immunohistochemistry stainings were performed as previously described (2). For a list of the antibodies used, see Supplemental Table 3. Images were taken with an Olympus BX-51 microscope, Olympus DP71 digital camera, and DP Controller software. Cell nuclei were counterstained with DAPI (Life Technologies, Grand Island, NY). Immunofluorescence images were acquired using an Olympus IX-71 confocal microscope and FluoView FV500/IX software. Histopathological analysis of PanIN lesions and quantification of pancreas size was performed as previously described (2).

Proliferation and Invasion Assays

Cell proliferation was measured using a CellTiter 96 AQ nonradioactive cell proliferation assay (Promega Corporation, Madison, WI) and in vitro invasion assays were performed using a Cell Invasion Assay Kit (Chemicon /Milipore International, Billerica, MA) as we have described previously (1).

Supplemental References:

(1) Wang, L., Heidt, D.G., Lee, C.J., Yang, H., Logsdon, C.D., Zhang, L., Fearon, E.R., Ljungman, M., and Simeone, D.M. 2009. Oncogenic function of ATDC in pancreatic cancer through Wnt pathway activation and beta-catenin stabilization. Cancer Cell 15:207-219.
(2) Hingorani, S.R., Wang, L., Multani, A.S., Combs, C., Deramaudt, T.B., Hruban, R.H., Rustgi, A.K., Chang, S., and Tuveson, D.A. 2005. Trp53R172H and KrasG12D cooperate to promote chromosomal instability and widely metastatic pancreatic ductal adenocarcinoma in mice. Cancer Cell 7:469-483.

Part Two: Supplemental Figure Legends

Supplemental Figure 1. (A) ATDC expression in pancreata harvested from 6-week-old control littermates (left) and CAG-ATDC mice (right). Scale bar: 50 µm. (B) Representative H & E images of pancreata from CAG-ATDC mice at different ages (1.5-12 months) reveals the development of acinar atypia without the formation of PanIN lesions or invasive PDA. Scale bar: 50 µm. (C) Number of PanINs was counted per 200X field (Mean±SE, n=5) (*p<0.05, AKC vs KC). (D) CAG-ATDC mice did not have alterations in organ size or cell death rate, however a slight increase in cell proliferation, compared to control littermates, was observed (*p<0.05, **p<0.01 vs WT).

Supplemental Figure 2. (A). Representative images of H&E staining of pancreata from wild type (WT), CAG-ATDC (ATDC), KC, and AKC mice at 1.5, 3, 6 and 8 months of age. (B) Alcian blue, trichrome, Ki67, MUC1 and CK19 staining of WT, KC, and AKC pancreatic tissues from 3 month old mice. (C) Representative H&E images of metastatic lesions that developed in 6 month old AKC mice. D) Representative H&E images of well, moderate, poorly differentiated or sarcomatoid regions of PDA in 6 month old AKC mice. Scale bar: 50 µm.

Supplemental Figure 3. (A) Representative Western blot showing p53 and p21 expression in AKC8 and14 cells 6 hours after treatment with the MDM2 antagonist, Nutlin-3 (10 µM). (B) Representative Western blot showing Smad4 expression in AKC8 and 14 cells 2 hours after TGFβ (10 ng/ml) treatment. β-actin served as loading control. (C) Immunofluorescent staining of Smad4 (red) and DAPI (blue) in AKC14 cells 2 hours after TGFβ (10 ng/ml) treatment. Scale bar: 10 µm.

Supplemental Figure 4. (A) ATDC is expressed in select cells in early PanIN lesions in KC mice. Scale bar: 10 µm. (B) qRT-PCR analysis of ATDC gene expression in KC and AKC mice at indicated ages. Data are expressed as mean ± SEM, *p<0.05 AKC vs KC, n=3.

Supplemental Figure 5. (A, B) Representative Western blot showing Kras and ATDC expression in KPC PDA (A) and Capan2 (B) cells with or without Kras shRNA. (C) Representative Western blot showing ATDC expression in KPC PDA and Capan2 cells expressing Kras shRNA with or without ATDC expression vector. (D, E) Cell growth and Invasion assays in KPC (D) and Capan2 cells (E) expressing Kras shRNA with or without an ATDC expressing vector. Data represents the mean ± SEM, *p<0.05 vs control shRNA (Cont shRNA), n=4.

Supplemental Figure 6. (A) Representative Western blot showing ATDC expression in AKC8, 14 and 17 cells. Normal pancreas (Cont Panc) served as a control. (B) qRT-PCR analysis of ATDC expression in AKC8, 14, and 17 cells expressing control or

ATDC shRNA1 and 2. (C) Schematic representation of ATDC shRNA-resistant vector. The ATDC shRNA target sequence (encoding amino acids 87-93) is indicated by capital letters. Mutations introduced by site-direct mutagenesis are indicated by red letters.

Supplemental Figure 7. (A) qRT-PCR analysis of E-cadherin, Snail1 and Zeb1 gene expression in KPC cells expressing control (Cont) or ATDC shRNA. Data represent mean ± SEM, *p<0.05 vs control shRNA (Cont), n=3. (B) Microscopic images of KPC PDA cells with or without ATDC shRNA.

Supplemental Figure 8. (A) Zeb1, (B) Snail1 and (C) E-cadherin expression in wild type (WT), CAG-ATDC, KC and AKC pancreatic tissues at the indicated ages. Scale bar: 50 µm.

Supplemental Figure 9. (A) ATDC (green), Snail1 (red) and DAPI (blue) co-immunofluorescent staining in early PanIN1 lesions from KC and AKC mice (3 m). Yellow indicates the co-localization of ATDC and Snail1. Arrows indicate mesenchymal-appearing ATDC and Snail1 double-positive cells in the stroma surrounding the PanIN lesion. Bar: 10 µm. (B) ATDC (green), Snail1 (red) and DAPI (blue) co-immunofluorescence in human PanIN1 lesions. Yellow staining indicates co-localization of ATDC and Snail1 in human PanIN cells. Bar: 10 µm. (C-D) KC mice (3 m) (C) and Human PanIN 1 lesion (D) demonstrating a mesenchymal-appearing cell in the stroma co-expressing ATDC and Zeb1 (arrow). Bar (yellow): 10 µm. Bar (white): 50 µm.

Supplemental Figure 10. (A) Knockdown of ATDC in AKC17 cells has no effect on GapDH mRNA levels. (B-C) qRT-PCR analysis of (B) Snail1 and (C) Zeb1 gene expression in AKC8, 14 and 17 cells expressing control or ATDC shRNA plus Snail1 or Zeb1 expression vectors. Data represent mean ± SEM, *p<0.05 vs control shRNA (Cont), n=3.

Supplemental Figure 11. qRT-PCR analysis of Snail1 (A), ATDC (B), β-catenin (C) and CD44 (D) gene expression in AKC8, 14 and 17 cells with or without Snail1 shRNA. Data represent mean ± SEM, *p<0.05 vs control shRNA (Cont), n=3. qRT-PCR analysis of Zeb1 (E), ATDC (F), β-catenin (G) and CD44 (H) gene expression in AKC8, 14 and 17 cells with or without Zeb1 shRNA. Data represent mean ± SEM, *p<0.05 vs control shRNA (Cont), n=3.

Supplemental Figure 12. (A) CD44 expression in WT, CAG-ATDC, KC and AKC mice 1.5, 6 and 12 months of age. CD44 staining was absent in pancreas from WT mice. Bar: 50 µm. (B) ATDC (green), CD44 (red) and DAPI (blue) co-immunofluorescence in PDA

from 6 month old AKC and 12 month old KC mice. Bar: 50 µm. (C) qRT-PCR analysis of CD44 gene expression in AKC8, 14 and 17 cells with or without ATDC shRNA +/- empty control or CD44 expression vector. Data represent mean ± SEM, *p<0.05 vs control shRNA (Cont), n=3. (D) qRT-PCR analysis of ATDC, β-catenin, CD44, Snail1 and Zeb1 gene expression in UM12 and UM59 cancer cells derived from primary human PDAs with control or ATDC shRNA. Data represent mean ± SEM, *p<0.05 vs control shRNA (Cont), n=4.

Supplemental Figure 13. (A) β-catenin expression in pancreata from WT, CAG-ATDC, KC and AKC (3 weeks old) mice prior to transformation. (B) ATDC (upper panel) and β-catenin (lower panel) expression in PanIN lesions and PDA from AKC mice. Bar: 50 µm.

Supplemental Figure 14. (A) co-IP with ATDC antibody using AKC14 or 17 cell lysates with or without ATDC shRNA1 or 2 expression, followed by western blotting using ATDC, Dvl2, or β–catenin antibodies. (B) Representative Western blot showing ATDC and β-catenin expression in AKC8, 14 and 18, KPC1 and 1, and KC1 and 2 cell lines. (C) Cell growth assays in AKC8, 14 and 17 cells expressing control or ATDC shRNA1 or 2, with or without β-catenin (β-cat) expression vectors. Data represents the mean ± SEM, *p<0.05 vs control shRNA (Cont shRNA), n=4.

Supplemental Figure 15. (A) ATDC (green), CD44 (red) Snail1 (purple) and DAPI (blue) co-immunofluorescence in AKC17 cells with or without ATDC shRNA. (B) ATDC (green), CD44 (red) Snail1 (purple) and DAPI (blue) co immunofluorescence in AKC17 cells with or without β-catenin shRNA. Bar: 50 µm.

Supplemental Figure 16. (A) Diagrammatic representation of the -1600 to +1 region of the mouse CD44 promoter. The putative binding sites for TCF-4 (TB) are indicated on the sequence. (B) qRT-PCR analysis of Cd44 gene expression in AKC8, 14 and 17 cells with or without ATDC shRNA plus β-catenin expression vector. Data represent mean ± SEM, *p<0.05 vs control shRNA (Cont), n=3.

Supplemental Figure 17. (A) Light (top panel) or fluorescence (low panel) images of mice revealing GFP expression in A^{LGSL}, A^{LGSL}C and A^{LGSL}KC mice. (B) Light (top panel) or fluorescence (low panel) images of pancreata demonstrating GFP expressed in A^{LGSL} pancreata, but not pancreata from in A^{LGSL}C and A^{LGSL}KC mice. (C) Western blot analysis of pancreatic tissue from A^{LGSL}, A^{LGSL}C and A^{LGSL}KC mice.
